# The WAVE2/miR-29/Integrin-β1 Oncogenic Signaling Axis Promotes Tumor Growth and Metastasis in Triple-negative Breast Cancer

**DOI:** 10.1158/2767-9764.CRC-22-0249

**Published:** 2023-01-31

**Authors:** Priyanka S. Rana, Wei Wang, Vesna Markovic, Justin Szpendyk, Ernest Ricky Chan, Khalid Sossey-Alaoui

**Affiliations:** 1 Department of Medicine, Case Western Reserve University, Cleveland, Ohio.; 2 MetroHealth Medical Center, Cleveland, Ohio.; 3 Case Comprehensive Cancer Center, Cleveland, Ohio.

## Abstract

Breast cancer is the most frequently diagnosed malignancy in women and the major cause of death because of its invasion, metastasis, and resistance to therapies capabilities. The most aggressive subtype of breast cancer is triple-negative breast cancer (TNBC) due to invasive and metastatic properties along with early age of diagnosis and poor prognosis. TNBC tumors do not express estrogen, progesterone, and HER2 receptors, which limits their treatment with targeted therapies. Cancer invasiveness and metastasis are known to be promoted by increased cell motility and upregulation of the WAVE proteins. While the contribution of WAVE2 to cancer progression is well documented, the WAVE2-mediated regulation of TNBC oncogenic properties is still under investigated, as does the molecular mechanisms by which WAVE2 regulates such oncogenic pathways. In this study, we show that WAVE2 plays a significant role in TNBC development, progression, and metastasis, through the regulation of miR-29 expression, which in turn targets Integrin-β1 (*ITGB1*) and its downstream oncogenic activities. Conversely, we found WAVE2 expression to be regulated by miR-29 in a negative regulatory feedback loop. Reexpression of exogenous WAVE2 in the WAVE2-deficient TNBC cells resulted in reactivation of *ITGB1* expression and activity, further confirming the specificity of WAVE2 in regulating Integrin-β1. Together, our data identify a novel WAVE2/miR-29/*ITGB1* signaling axis, which is essential for the regulation of the invasion-metastasis cascade in TNBC. Our findings offer new therapeutic strategies for the treatment of TNBC by targeting WAVE2 and/or its downstream effectors.

**Significance:**

Identification of a novel WAVE2/miR-29/*ITGB1* signaling axis may provide new insights on how WAVE2 regulates the invasion-metastasis cascade of TNBC tumors through the modulation of *ITGB1* and miR-29.

## Introduction

Breast cancer is the most common cause of cancer in women in United States accounting for 31% of all estimated new cancer cases and 15% of all cancer-related fatalities (1). Among its variants, triple-negative breast cancer (TNBC) is considered the most aggressive disease that affects many women, due to its early invasive and metastatic properties and absence of targeted therapies that are FDA approved (2–4). Because TNBC tumors do not express cell surface receptors estrogen receptor (ER), progesterone receptor (PR), and Her2 which can be targeted with hormonal and antibody treatments, patient with TNBC are left with limited treatment options in the form of cytotoxic chemotherapy with dismal response and rapid recurrence due to the acquisition of resistance. Several oncogenes orchestrate a myriad of signaling reactions that contribute toward tumor development, invasion, and metastasis (5). Among the WAVE family of proteins (6, 7), WAVE2 is known to be associated with pathogenesis of several cancers and recently has been a topic of great interest in cancer invasion and metastasis (8). Owing to its critical role in actin cytoskeleton remodeling, WAVE2 mediates cell motility, migration, and cancer invasion (9–13), which are among the critical properties that promote metastasis when abnormally upregulated in several malignancies (14). Although, several studies and clinical data revealed that WAVE2 is enhanced in different cancers, the molecular mechanisms by which it exerts its oncogenic properties in specific cancer types still need to be investigated. With this research, we have shown how WAVE2 is involved in the invasion-metastatic cascade of breast cancer, both *in vitro* and *in vivo*.

Cancer cell invasion, progression, and metastasis are driven by interactions between tumor cells and their microenvironment (15, 16). The extracellular matrix (ECM) is a major structural component of tumor microenvironment. ECM is a noncellular component of tissue that is composed of cross-linked macromolecules such as collagens, proteoglycans, and glycoprotein that form a dynamic scaffold and provide physical and chemical stimuli to mediate cancer progression and metastasis (17). ECM macromolecules also serve as ligands for cell surface integrins (18), which upon activation, trigger a signaling cascade that regulate a myriad of activities ranging from cell adhesion, spreading, as well as cell migration and invasion (19). While the literature is filled with studies on the regulatory mechanisms of integrins activity, the role of WAVE2 as a potential regulator of integrins has not been reported before, and, as such, is the focus of this study.

Research in the last decade identified the role of epigenetics, such as DNA methylation, histone modifications, abnormal expression of certain noncoding RNAs, and chromatin remodeling, as potential drivers of breast cancer development and progression (20). miRNAs are a type of conserved noncoding RNAs that are known for modulating the expression of several genes that regulate diverse and complex cellular pathways such as cell development, growth, proliferation, and motility and, thus possess the potential to also regulate the major hallmarks of cancer by modulating several oncogenes (21, 22). miRNAs have also been established as either oncogenes or tumor suppressors to regulate tumor progression and metastasis (23). The miR-29 family of miRNAs consists of miR-29a, miR-29b, and miR-29c that regulate several biological processes such as intracellular signaling (24), epigenetic modifications (25), cell proliferation (26), and cell motility (27). In this study, we report novel findings that describe how a negative regulatory feedback loop between WAVE2 and miR-29 regulates Integrin-β1 (*ITGB1*) expression and activity, and in the run regulates major hallmarks of TNBC tumors. Our data show that loss of WAVE2 expression results in increased miR-29 levels, which, in turn inhibits *ITGB1* expression through binding to its 3′-UTR (untranslated region). Conversely, overexpression of miR-29 suppresses the expression of WAVE2 by also targeting its 3′-UTR. This WAVE2/miR-29/*ITGB1* signaling axis is critical for the regulation of tumor growth and metastasis of TNBC tumors. We further identified a potential mechanism whereby WAVE2 activates miR-29, possibly through the regulation of DGCR8, a major mediator of miRNA biogenesis, which may explain, in part, the negative feedback loop between WAVE2 and miR-29. Importantly, our *in vitro* and mouse preclinical findings are supported by human clinical data where increased expression of WAVE2 and *ITGB1*, and decreased expression levels of miR-29 and DGCR8 correlate with poor disease outcome in human patients with breast cancer tumors. Together, our data identified a novel WAVE2/miR-29/*ITGB1* signaling axis that regulates the invasion-metastasis cascade in breast cancer. Thus, successful characterization of the WAVE2 signaling pathway in cancer cell invasion and metastasis could serve as a novel approach for developing new therapeutic strategies for targeting TNBCs.

## Material and Methods

### Cell Culture

TNBC 4T1, MDA-MB-231, and MDA-MB-468 cell lines and HEK 293 cells were obtained from ATCC. All the cell lines were maintained according to the manufacturer's protocols. No authentication was performed in the lab, because we relied on the manufacture's quality control statement. Cell lines were routinely (on an average of 9 to 12 months) tested for *Mycoplasma* contamination. Cells were cultured at early passages (no more than 10), and were passaged in culture no more than five times before a new vial is being thawed. Generation of WAVE2- and *ITGB1*-deficient cells was used through electroporation of breast cancer cells with single-guide RNA (sgRNA)/Cas9 mixes (Synthego), according to the manufacturer's instructions. For each human or mouse gene, a pool of three verified sgRNAs was used (Synthego). Scrambled sgRNAs (Synthego) were used a negative control. Efficient and stable gene knockout (KO) was verified by Western blot (WB) analysis. In some instances when the knockout efficiency is less than 80%, a second round of sgRNA delivery is performed. Generation of breast cancer cells overexpressing miR-29 cells were generated by nucleofection delivery of miR-29 expressing vector or its empty control vector, and stable clones were selected for by adding G418 (2 mg/mL) to the complete culture medium for 2 weeks as described previously in ref. 28. The HA-tagged WAVE2-expressing vector (pCS-HA-WAVE2) was kind gift from Dr. Alexis Gautreau (Institut Curie, Paris, France). Transient transfection of W2-KO-MDA-MB-231 cells with HA-WAVE2 and of HEK293 cell with dual luciferase pmiR-Glo plasmid and miR-29–expressing plasmids were performed using Lipofectamine 3000 reagent according to the manufacturer's instructions and as described previously in ref. 29. All the cell lines were grown in complete culture medium: DMEM culture medium supplemented with 10% FBS and 5% Antibiotics (Penn/Strep). Transfected cell lines were selected in complete culture medium with selection antibiotic.

### Antibodies

The following antibodies were used: rabbit antibodies against WAVE2 (1:1,000, Cell Signaling Technology, catalog no. 3659), Integrin β1/ CD29 (GeneTex, catalog no. GTX128839), and DGCR8 (Abcam, catalog no. Ab191875); mouse antibodies against β-actin (1:5,000, Sigma), WAVE1 (1:200, Santa Cruz Biotechnology, catalog no. Sc-136120), and WAVE2 (1:1,000, Santa Cruz Biotechnology, catalog no. Sc-373889). Rabbit anti FAK (1:1,000, Invitrogen, catalog no. UB281522), rabbit anti-phospho-FAK (1:1,000, Abcam, catalog no. ab81298), rabbit anti Src (1:1,000, Cell Signaling Technology, catalog no. 2109S), rabbit anti phospho-Src (1:1,000, Cell Signaling Technology, catalog no. 6943S), goat horseradish peroxidase–conjugated anti-mouse IgG and goat horseradish peroxidase–conjugated anti-rabbit IgG were from Bio-Rad (1:2,000). ECL reagent was from Thermo Fisher Scientific. For immunoblotting, primary antibodies were made in 5% BSA and secondary antibodies were made in 5% non-fat dry milk (NFDM).

### Western Blotting

Immunoblotting analyses were performed according to standard protocols and as described previously in ref. 30. ChemiDoc MP Imaging system (Bio-Rad) was used for image acquisition of developed gels. Band intensity of proteins targeted were quantitatively analyzed by ImageJ software according to the parameters described in ImageJ user guide (http://rsbweb.nih.gov/ij/docs/guige/146.html, accessed on July 2, 2021).

### Flow Cytometry Analyses

MDA-MB-231 and their derivatives W2KO, miR-29–expressing, and ITGB1KO cells were detached by trypsinization and washed with FACS staining buffer (PBS with 5% BSA). Cells at a density of 5 × 10^5^/mL were stained with conjugated PE Mouse Anti-Human CD-29 antibody (BD Pharmingen, catalog no. 555443) for 30 minutes followed by washing and resuspending the cells in 250 μL FACS staining buffer. Anti-Integrin β1 Antibody, activated clone HUTS-4 (Sigma, catalog no. MAB2079Z) was used to detect the activation of *ITGB1*. As a positive control for integrin activation, 0.5 mmol/L MnCl_2_ was used to treat the cells for 10 minutes at 37°C. Processed cells were then run through FACSAria and all the data were analyzed using FlowJo software. Data were corrected for any nonspecific signals and the resultant fluorescence intensities were plotted graphically.

### Semiquantitative and qRT-PCR

TRIzol reagent (Invitrogen) was used to extract total RNA from cancer cell lines according to the manufacturer's instructions. qRT-PCR was performed, as described previously in ref. 30. qPCR Primer Assays for miR-29 were obtained from Qiagen. U6 was used a negative control.

### RNA Sequencing and Biostatistical Analyses

Control and WAVE2-KO MDA-MB-231 and MD-MB468 cells were grown to near confluency (80% to 90%) in 10-cm tissue culture dishes in DMEM supplemented with 10% FBS. Early passages were used for RNA extraction and subsequent RNA sequencing (RNA-seq) analyses as described previously (28).

#### TruSeq Total RNA Stranded Library Preparation and Sequencing

RNA quality for each sample assessed using Agilent's Bioanalyzer 2100 RNA Nano 6000 reagent kit, before library preparations. RNA integrity number value higher than 8, indicating minimal sample degradation, was required. Illumina TruSeq Total RNA Stranded Library Preparation Kit was used to generate a range of total RNA from 100 ng to 1 μg. Library preparation was performed according to the manufacturer's instructions. The final cDNA library size distribution was checked using a Bioanalyzer DNA High Sensitivity assay, followed by analysis Qubit to determine the concentration of each sample. Samples are then pooled and the accurate concentration for the pool is determined using the Kapa Library Concentration Kit.

#### Sequencing

Illumina HiSeq 2500 set in Rapid Run mode was used for sequencing of up to 12 RNA samples. We used the paired end 100 cycle kit (meaning that 100 cycles will be used for the forward and reverse directions so a total of 200 cycles is used) to generate approximately 30–40 million reads per sample, in biological replicates (three per condition) to be able to yield statistically significant results. Sequencing results are delivered as FASTQ files which can then be aligned and analyzed using bioinformatics.

#### RNA-seq Analysis Methods

Sequencing reads generated from the Illumina platform were assessed for quality using FastQC. The reads were trimmed for adapter sequences using TrimGalore. For RNA-seq, reads that passed quality control were then aligned to the human reference genome (GRCh38) using the STAR aligner41. The alignment for the sequences was guided using the GENCODE annotation for GRCh38. The aligned reads were then analyzed for differential expression using cufflinks42, a RNA-seq analysis package which reports the fragments per kilobase of exon per million fragments mapped for each gene. The 12 samples were analyzed in four groups of three (WT, W3, K2, and DKO) and differential expression analysis was performed in a pairwise manner. Differential genes were identified using a significance cutoff of FDR < 0.05. These genes were then subjected to gene set enrichment analysis (Broad Institute) to determine any relevant processes that may be differentially overrepresented for the conditions tested.

### Cell Adhesion and Spreading Assays

For adhesion and spreading assays, 5 × 10^5^ cells were seeded on round 1.5 mm coverslips (Electron Microscopy Sciences) precoated with 10 mg/mL laminin (Sigma), 10 mg/mL fibronectin (Sigma), or five times diluted growth factor–reduced Matrigel (Corning). Cells were allowed to adhere for 30 minutes before fixation or spread overnight on these polymers. A total of 4% paraformaldehyde (PFA) dissolved in PBS was used to fix the cells for 20 minutes at room temperature followed by 3X wash and permeabilization with 0.5% Triton-X for 10 minutes at room temperature. The spread cells were then washed and probed with phalloidin-643 (1:30, Invitrogen, catalog no. A30107) for 60 minutes and mounted on slides using the ProLong Gold antifade reagent with DAPI (Invitrogen, catalog no. P36931). For adhesion assay, fixed and permeabilized cells were directly mounted on slides using the mounting medium. Cell spreading was assessed by collecting several images of cells and quantified by marking an area around the cells in ImageJ and comparing the surface area between different groups of cells. For adhesion assay, attached cells stained with DAPI were imaged and several fields were used for counting attached cells using ImageJ.

### Plasmid Construction, Site-directed Mutagenesis, and 3′-UTR Dual Luciferase Reporter Assays

The nucleotide sequence flanking the miR-29 seed sequence in the 3′-UTR of WAVE2 and *ITGB1* from human genomic DNA was amplified by PCR, then subcloned into the pmirGlo vector (Promega) downstream of the firefly luciferase, as described previously in refs. 29, 31. The correct sequence and orientation of all the inserts was verified by sequencing. To generate mutations in the seed sequence, the QuickChange site-directed mutagenesis kit was used where the seed sequence recognized by miR-29 in *ITGB1* and WAVE2 was scrambled. The mutated sequence was also verified by sequencing. pmirGlo reporter plasmids were transfected with Lipofectamine-3000 (Invitrogen) in HEK-293 cells as described previously. Cells were collected after 48 hours for assay using the dual luciferase reporter assay system (Promega).

### *In Vitro* Tumorsphere Growth and Invasion Assays

Assays for three-dimensional (3D) tumorsphere growth and invasion were performed as described previously (32). For 3D single-tumorsphere formation, MDA-MB-231 cells and their derivative W2KO cells were seeded on 96-well ultralow attachment (ULA) plate at a density of 1.0 × 10^3^ cells per well and centrifuged for 10 minutes at 125 × *g* at room temperature. The cells were the imaged every 2–3 days for 11 days on a Leica CMi1 microscope to monitor the growth of 3D-tumorsphere formation. For the invasion assays, the cells were supplemented with 90 μL of Matrigel (dissolved at 1:1 in complete medium) on top at the end of day 3 and the plate was imaged every 48 hours. to monitor the invasive potential of the spheroids. For 3D-multiple tumorsphere formation assay, cells were plated in a 6-well dish at a density of 2 × 10^3^/well precoated with polyhema. The dish was imaged every 48 hours for 10 days with a Leica DMi1 microscope to monitor the cell's potential to form multiple tumorsphere in 3D.

### Animal Experiments

MDA-MB-231, and their derivatives W2KO, miR-29–overexpressing, ITGB1KO cells were implanted at a density of 10^6^ cells/injection into the mammary fat pads in both sides of female NSG mice***.***Mice were monitored for tumor growth twice for 8 weeks and tumor volume was measured with digital Vernier calipers. For lung colonization assay, cells at a density of 100,000 suspended in 100 μL sterile PBS were injected via 28-guage needle into the tail veins of 6 to 8 weeks old female NSG mice. Mice were sacrificed 5 weeks later, and the recovered lungs were imaged under dissecting microscope. Lung metastasis nodules were counted for the images and results were plotted as average number of metastatic foci per lobe. Similar experiments were carried out the 4T1 cells and their W2KO derivatives (100,000 cells/injection in the mammary fat pads and 50,000 cells/injection in tail vein of Balb/C mice).

### Immunofluorescence

For immunofluorescence (IF), cells were processed as described previously (33). In brief, cells were fixed with 4% PFA in PBS for 20 minutes at room temperature followed by three PBS washes, permeabilization with 0.5% Triton-X for 10 minutes at room temperature and three PBS washes. Cells were then blocked with 5% donkey serum (dissolved in PBS) for 60 minutes and probed with primary antibodies diluted in 5% donkey serum overnight at 4°C. The following day cells were washed with PBS and probed with a secondary antibody for 1–2 hours at room temperature, washed and mounted on slides using the ProLong Gold antifade reagent with DAPI (Invitrogen, catalog no. P36931). The following antibodies were used for IF: Rabbit anti-WAVE2 (Cell Signaling Technology, catalog no. 3659) and mouse anti-Rabbit anti-*ITGB1*/CD29 (GeneTex, catalog no. GTX128839).

### Oligonucleotide Sequences

Sequences of the oligonucleotide primers used for genomic PCR, RT-PCR, those used to amplify WAVE2 and *ITGB1* 3′-UTR harboring miR-29 seed sequences, as well as the primers used for mutagenesis were from IDT and are listed in Supplementary Table S1.

### Statistical Analysis

All experiments were performed in triplicates and were analyzed using the Student *t* test. Error bars represent SEM. The two-tailed significance levels for equal means and equal variances were assumed for the two populations and results were considered significant at *P* < 0.05.

### Study Approval

All studies involving animals were performed under the protocols approved by Institutional Animal Care and Use Committee. The animal studies were conducted in accordance with the guidelines and regulations that were approved by MetroHealth Medical Center, Case Western Reserve University (Cleveland, OH), and NIH. For all the following animal experiments 6 to 8 weeks old female NSG or Balb/C mice from Jackson Laboratory were used. Experimental mice were routinely observed twice a week according to the protocol.

### Data Availability Statement

Data were generated by the authors and included in the article*.* The data generated in this study are available within the article and its Supplementary Data.

## Results

### WAVE2 is Highly Expressed in Basal Subtype of Breast Cancer Tumors and is Associated with Poor Survival and Worst Patient Outcomes

**FIGURE 1:**
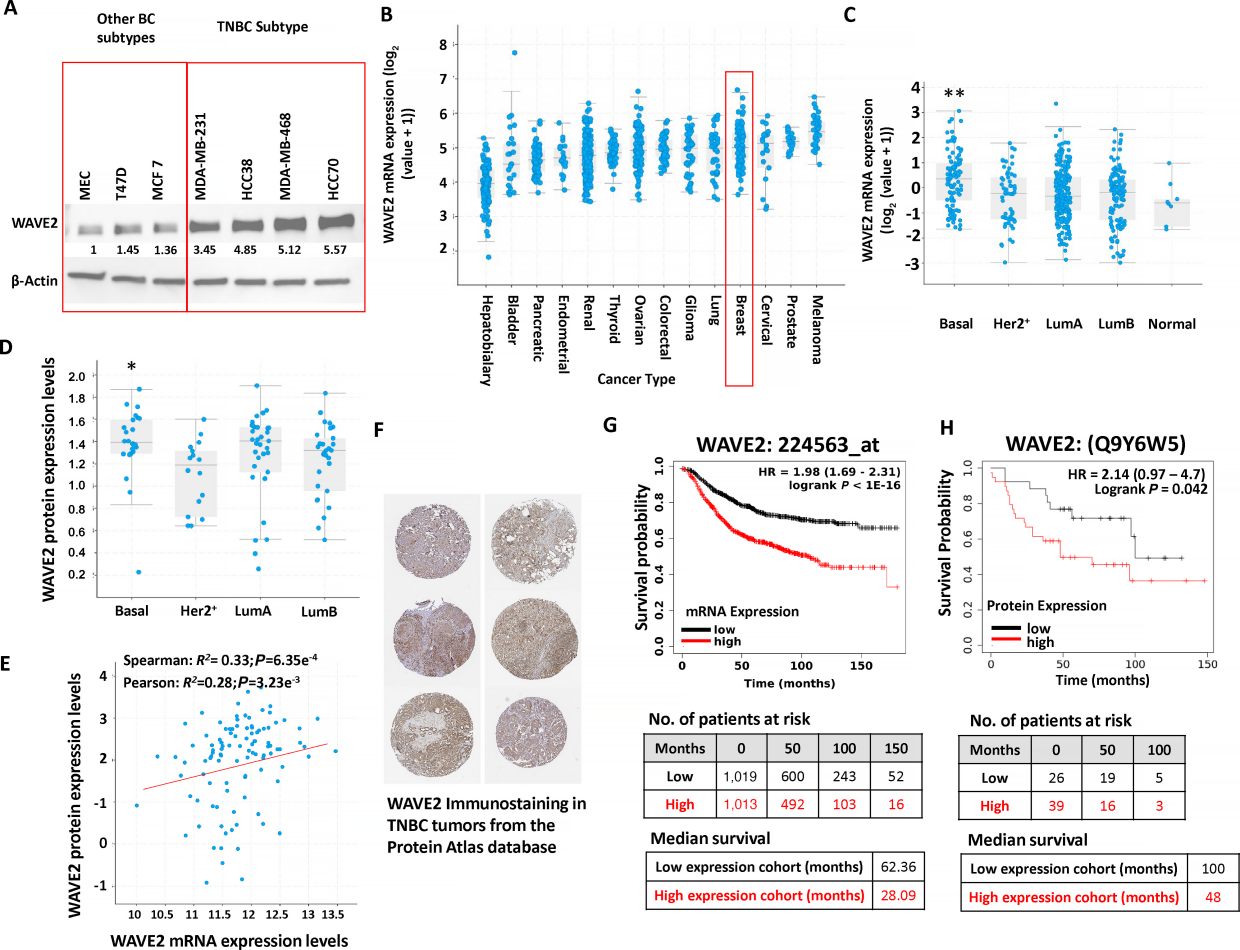
WAVE2 is highly expressed in basal subtype of breast cancer tumors and is associated with poor survival and worst patient outcomes. **A,** Representative WB analysis of protein lysates from different breast cancer cell lines probed with the indicated antibodies. β-Actin was used for loading control. **B,** mRNA expression levels of WAVE2 in different cancer datasets derived from the cBioPortal platform. Data shown are representative of three replicates. **C,** Quantification of WAVE2 mRNA expression levels based on log_2_ RSEM values batch normalized from Illumina HiSeq RNA-seq data by breast cancer subtype in the breast cancer BRCA patient data from TCGA PanCancer Atlas. Expression levels of WAVE2 are significantly higher (**, *P* < 0.01, Wilcoxon) in the basal (TNBC) subtype when compared with other breast cancer subtypes. **D,** Quantification of WAVE2 protein expression levels by breast cancer subtype in the breast cancer BRCA patient data from TCGA PanCancer Atlas. Expression levels of WAVE2 are significantly higher (**, *P* < 0.05, Wilcoxon) in the basal (TNBC) subtype when compared with other breast cancer subtypes. **E,** Correlation between WAVE2 mRNA and protein levels in tumors of patients with breast cancer from TCGA PanCancer Atlas dataset. **F,** Representative IHC staining pictograms of human TNBC tumors stained with anti-WAVE2 antibody from the Protein Atlas Database. KM plot correlating survival of patients with breast cancer with WAVE2 mRNA (**G**) and protein (**H**) expression levels. High WAVE2 expression levels correlate with poor survival probability in patients with breast cancer (*P* < 1e^−16^ for mRNA and *P* = 0.042 for protein). Number of patients at risk and median survival in the low and high WAVE2 cohorts are also shown.

Our previous studies have extensively reported on the role of WAVE3 in mediating tumor progression and metastasis in breast cancer (reviewed in refs. 6 and 34). The role of WAVE2, a close relative of WAVE3, in the pathogenesis of breast cancer, has, however, not been investigated. To initiate this study, we began by assessing WAVE2 protein expression levels in a series of breast cancer cell lines representing different breast cancer subtypes (Fig. 1A). While we found WAVE2 to be expressed in every cell line, including mouse mammary epithelial cells, WAVE2 expression levels were significantly higher in cell lines of aggressive basal subtype as compared with their less aggressive counterparts (Fig. 1A). To confirm this observation, we interrogated the cancer datasets from the cBioPortal and found breast cancer among the pan-cancer cohort where WAVE2 is predominantly highly expressed (Fig. 1B). Further interrogation of The Cancer Genome Atlas (TCGA) PanCancer breast cancer cohort, which contains clinical information on more than 1,000 patients with breast cancer, showed WAVE2 mRNA expression levels to be significantly (*P* < 0.01) higher in the basal (TNBC) breast cancer subtype, as compared with their Her2^+^ and luminal counterparts or to normal breast tissue (Fig. 1C; Supplementary Fig. S1A and S1B). WAVE2 protein expression levels were also found to be significantly (*P* < 0.05) highly expressed in human tumors of TNBC subtypes, when compared with their Her2^+^ and luminal counterparts (Fig. 1D). Accordingly, a significant positive correlation was observed between mRNA and protein levels of WAVE2 in TCGA PanCancer breast cancer cohort (Fig. 1E). In addition, interrogation of the Protein Atlas database (https://www.proteinatlas.org/ENSG00000158195-WASF2/pathology/breast±cancer), that contains WAVE2 IHC data on human breast cancer tumors, also showed a significant increase of WAVE2 staining in cancer cells, compared with the stroma (Fig. 1F). Next, we interrogated the breast cancer Kaplan Meier (KM) plotter (https://kmplot.com/analysis) cohort, which contains clinical information on approximately 5,000 patients with breast cancer, and found a very significant (*P* < 1e^−16^) correlation between elevated WAVE2 mRNA (Fig. 1G) or protein (Fig. 1H) expression and reduced survival probability. In this cohort, patients with breast cancer with high WAVE2 expression levels in their tumors had worst clinical outcomes when compared with patients with low WAVE2 levels, and patients with high tumor WAVE2 mRNA levels have an average reduced survival of 36 months (Fig. 1G), while those with high tumor WAVE2 protein levels have an average reduced survival of 52 months (Fig. 1H) when compared with patients with low tumor WAVE2. This inverse correlation remains significant when accounting for only ER^−^ (Supplementary Fig. S1C) or ER^−^/PR^−^ (Supplementary Fig. S1D). Thus, these findings support the hypothesis of WAVE2 as a promoter of breast cancer aggressiveness and warrant further investigations.

### Loss of WAVE2 Inhibits the Oncogenic Behavior of TNBC Cell Lines *In Vitro*

**FIGURE 2:**
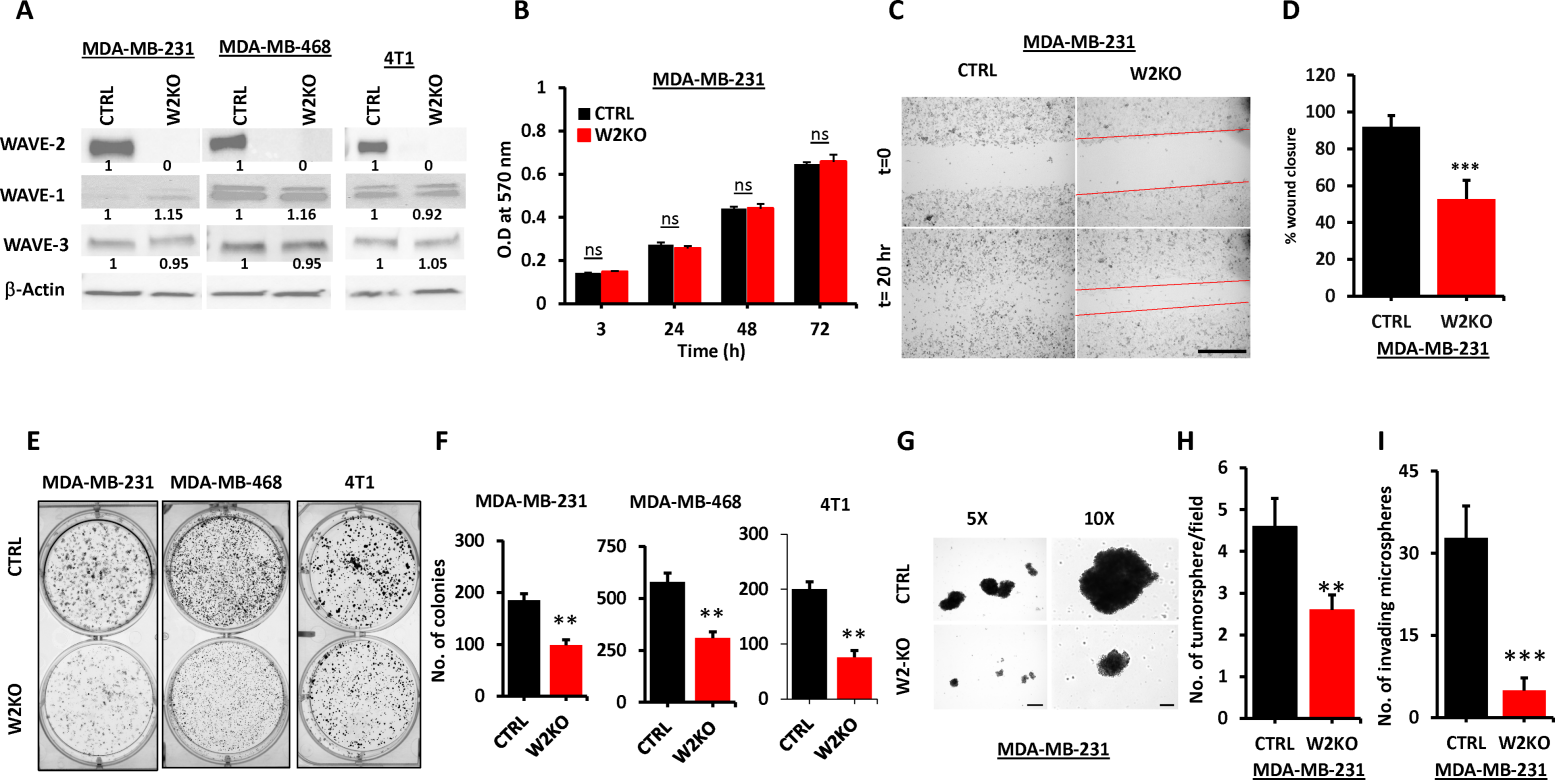
Loss of WAVE2 inhibits the oncogenic behavior of TNBC cell lines *in vitro*. **A,** Representative WB analysis of protein lysates from MDA-MB-231, MDA-MB-468, and 4T1 breast cancer cell lines and their WAVE2-KO derivatives probed with the indicated antibodies. β-Actin was used as loading control. The numbers under the WB bands represent the fold change of the signal with respect to the CTRL band after normalization to the β-Actin signal. **B,** Cell proliferation of MDA-MB-231 cells (CTRL) and their WAVE2-deficient derivatives (W2KO) over 3 days. **C,** Representative micrographs of wound healing assays of confluent cell cultures of MDA-MB-231 (CTRL) cells, their WAVE2-deficient derivatives (W2-KO) that were induced to migrate into scratch wounds in confluent monolayers over 20 hours. Scale bar: 500 μm. **D,** Quantification of the remaining open wound (open area) at 20 hours from 12 different wounds was measured and plotted as the percentage of the wound at time zero for CTRL cells. **E,** Representative images of colony formation of the indicated CTRL and W2-KO TNBC cell lines. **F,** Quantification of the number of colonies. **G,** Representative micrographs of tumorspheres from CTRL and W2-KO MDA-MB-231. Scale bar: 250 μm for 5x and 100 μm for 10x. Tumorspheres were grown in a 96-well ULA plates and Matrigel (2.5 v/v) was added to the tumorsphere cultures at day 3 and images were captured using Incucyte for 14 days. **H,** Quantification of the number of tumorspheres. **I,** Quantification of the number of invading microspheres. Data are the means ± SD (*n*  =  3; ** and ***, *P* < 0.01; Student *t* test). Data shown are representative of three replicates.

To enable a comprehensive characterization of phenotypic consequences of targeting WAVE2 signaling *in vitro*, we used nucleofection and CRISPR/Cas9 to generate WAVE2-KO in human MDA-MB-231 and MDA-MB-468 and murine 4T1 TNBC cells (Fig. 2A), by delivering a pool of three verified WAVE2-sgRNAs (Supplementary Table S1) in complex with Cas9 protein into these TNBC lines. We generated pools of cell populations of WAVE2-KO for MDA-MB-231, MDA-MB-468, and 4T1 cells. Nontargeting sgRNAs served as controls (CTRL). This approach resulted in almost complete loss of WAVE2 expression (W2KO) in all three cell lines, without affecting expression of WAVE1 and WAVE3 (Fig. 2A). Loss of WAVE2 did not affect cell proliferation in two-dimensional (2D) culture (Fig. 2B). Next, cell migration was assessed in the control (CTRL) MDA-MB-231 cells and their W2KO derivatives using the 2D wound healing assay, and the extent of wound closure was compared between the two groups after 20 hours. Approximately 90% of the wound was closed in the control group (CTRL) whereas only 50% of wound closure was achieved in the W2KO cells (Fig. 2C and D). We also used colony formation assay, which is a hallmark of transformed cells, to assess oncogenic potential, and found that the loss of WAVE2 significantly (*P* < 0.01) inhibits colony formation of all three TNBC cell lines: MDA-MB-231, MDA-MB-468, and 4T1 (Fig. 2E and F). In addition, we used 3D single and multiple tumorsphere growth (Fig. 2G and H), as well as tumorsphere-invasion assays (Fig. 2I; Supplementary Fig. S2) to assess the effect of loss of WAVE2 on cancer cell growth and invasion in 3D conditions. We found loss of WAVE2 in MDA-MB-231 cells significantly (*P* < 0.01) inhibited the growth and number of tumorspheres (Fig. 2G and H) as compared with the control cells. Moreover, the loss of WAVE2 expression significantly (*P* < 0.001) inhibited the Matrigel invasion of MDA-MB-231 cells (Fig. 2I; Supplementary Fig. S2). Thus, our *in vitro* findings confirm the involvement of WAVE2 in the activation of the oncogenic activities of TNBC cell lines.

### Loss of WAVE2 Inhibits Tumor Growth and Metastasis *In Vivo*

**FIGURE 3:**
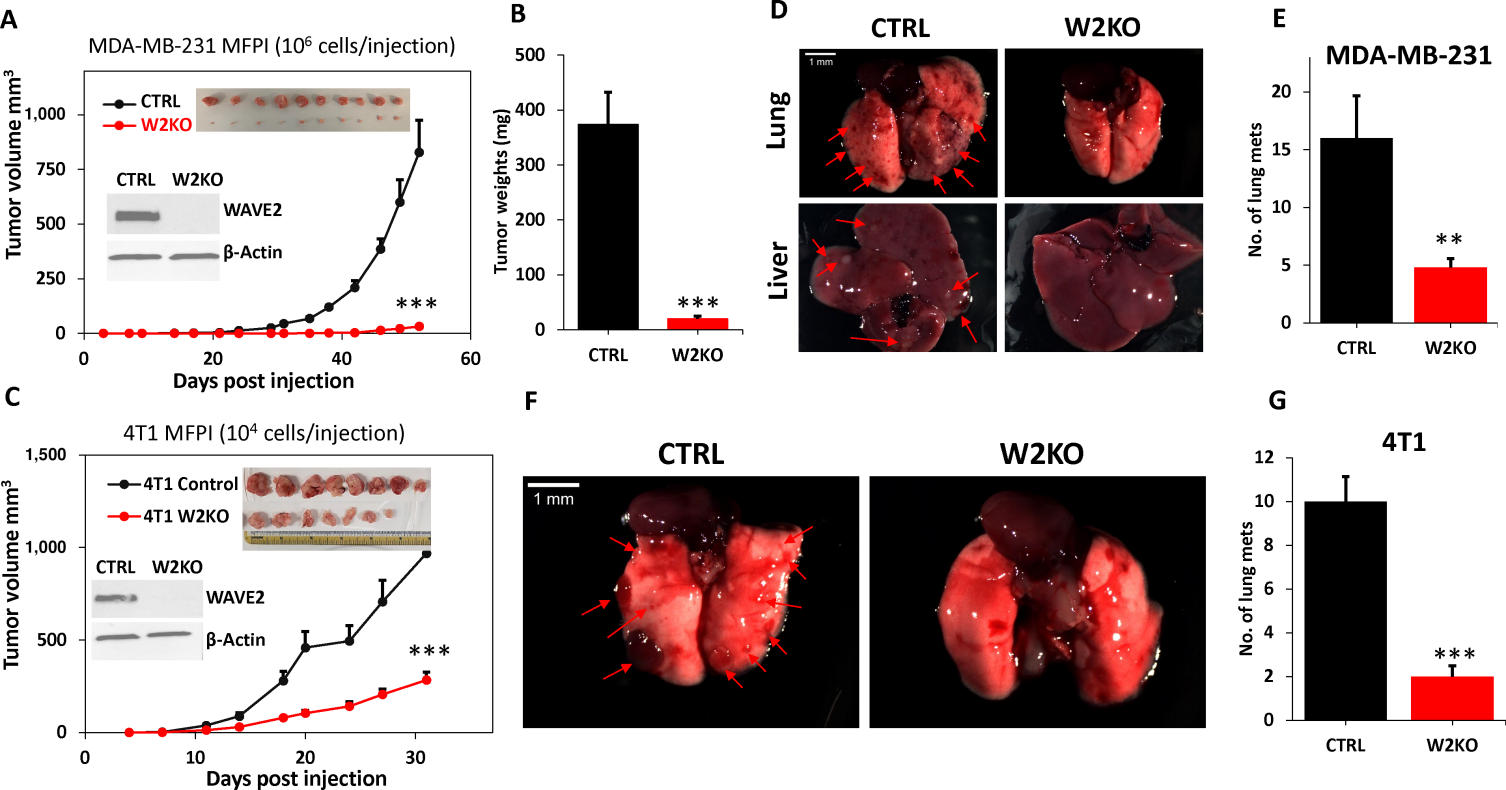
Loss of WAVE2 inhibits tumor growth and metastasis *in vivo*. **A,** Quantification of volume of tumors derived from implantation of CTRL or WAVE2-KO MDA-MB-231 cells into the mammary fat pads of NSG mice. The inserts show WBs confirming W2-KO in MDA-MB-231 cells before mice injections, as well images of the resulting tumors from each group. **B,** Quantification of tumor weights from the experiment described in A. **C,** Quantification of volume of tumors derived from implantation of CTRL or WAVE2-KO 4T1 cells into the mammary fat pads of Balb/C mice. The inserts show WBs confirming W2-KO in 4T1 cells before mice injections, as well images of the resulting tumors from each group. **D,** Images of lungs (top) and livers (bottom) from NSG mice injected via the lateral tail veins with CTRL or W2KO MDA-MB-231 cells. **E,** Quantification of lung metastasis foci from the corresponding experiment. **F,** Images of lungs from Balb/C mice injected via the lateral tail veins with CTRL or W2KO 4T1 cells. **G,** Quantification of lung metastasis foci from the corresponding experiment. ** and ***, *P* < 0.01; Student *t* test.

**FIGURE 4:**
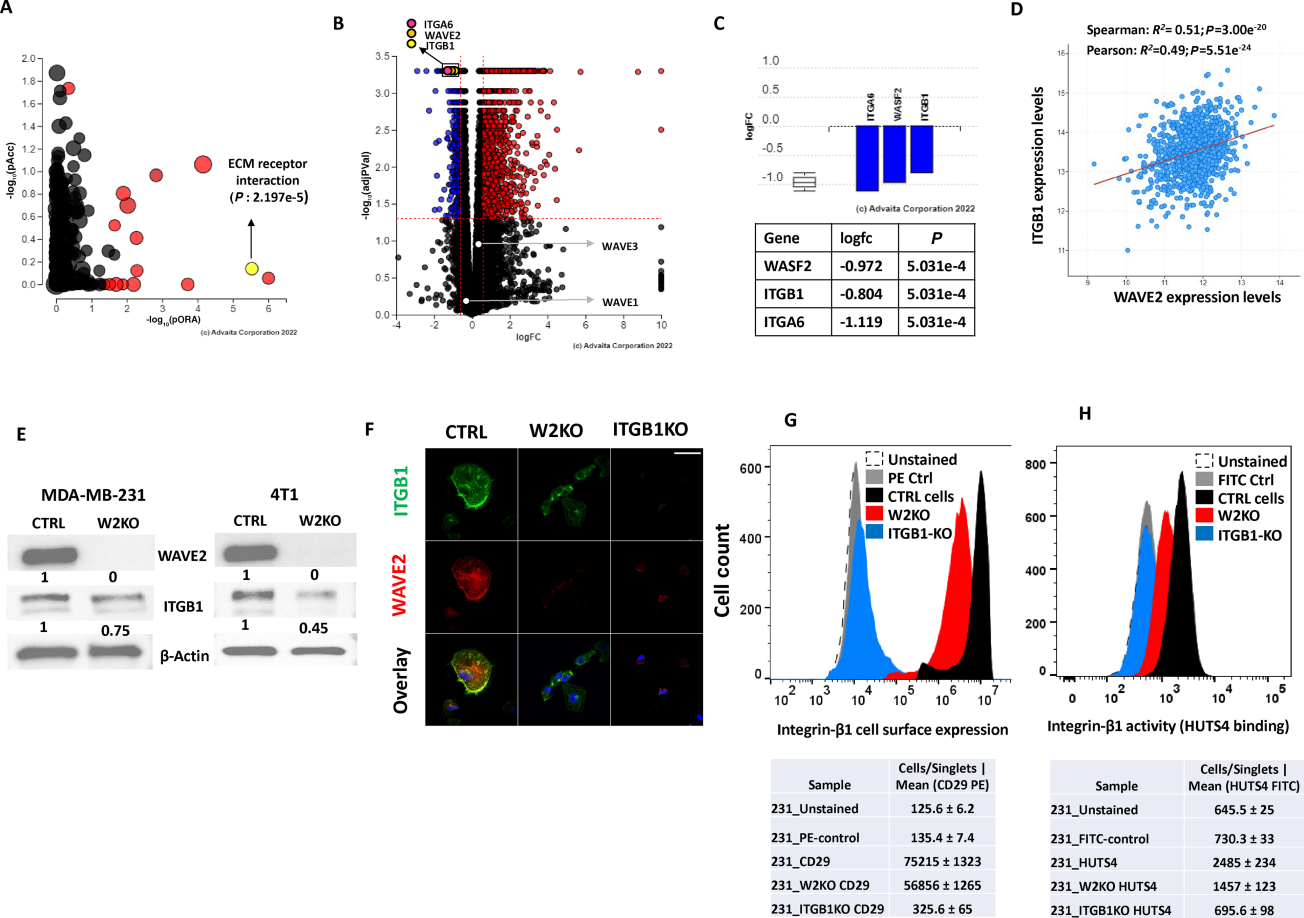
Loss of WAVE2 inhibits *ITGB1* expression and activity. **A,** Pathway analysis of the RNA-seq generated from CTRL and W2-KO MDA-MB-231 cells. Each dot represents a signaling pathway. The red dots represent the pathways that are significantly (*P* < 0.05) differentially regulated between the CTRL and the W2-KO groups. The black blots represent nonsignificantly differentially regulated pathways. The ECM receptor interaction pathway is shown with a yellow dot (*P* = 2.197e^−5^). **B,** Volcano plot from the RNA-seq analysis of the differentially expressed genes between CTRL and W2-KO MDA-MB-231 cells. The *X*-axis represents the log_2_ fold change in expression levels and the *Y*-axis shows the *P* values. Red dots: upregulated genes; Blue dots: downregulated genes; Black dots: no significant change. The dots of the genes of interest are labeled. **C,** Bars and values representation of the log_2_ fold change in expression levels of WAVE2, *ITGB1*, and ITGA6 derived from the RNA-seq data. **D,** Correlation of expression levels between WAVE2 and *ITGB1* in human breast cancer specimens from the cBioPortal breast cancer datasets (*P* < 0.01). **E,** Representative WB analysis of protein lysates from CTRL and W2-KO MDA-MB-231and 4T1 breast cancer cell lines probed with the indicated antibodies. β-Actin was used for loading control. The numbers under the WB bands represent the fold change of the signal with respect to the CTRL band after normalization to the β-Actin signal. **F,** Confocal microscopy images of IF staining of CTRL, W2-KO, and *ITGB1*-KO MDA-MB-231 cells that were stained for WAVE2 (red) or *ITGB1* (green). Nuclei were counterstained with DAPI. Scale bar: 50 μm. Representative histograms of flow cytometry analyses for cell surface expression of *ITGB1* (**G**) and activity: HUTS4 binding (**H**) of CTRL, W2-KO and *ITGB1*-KO MDA-MB-231 cells. A shift to the right indicates increased expression or activity. Data shown are representative of three replicates.

To determine the effects of the loss of WAVE2 on tumor growth *in vivo*, mammary fat pads of NSG mice were inoculated with control or W2KO MDA-MB-231 cells and tumor growth was assessed over 8 weeks. We found that the loss of WAVE2 significantly (*P* < 0.001) inhibited the growth of primary tumors, as determined by tumor volume (Fig. 3A; Supplementary Fig. S3A) and tumor weight (Fig. 3B). Similarly, the loss of WAVE2 in the 4T1 cells also significantly (*P* < 0.01) delayed tumor growth in Balb/C mice (Fig. 3C; Supplementary Fig. S3B). These resulted were replicated with MDA-MB-468, another TNBC cell line (Supplementary Fig. 4). Control and W2KO MDA-MB-231 and 4T1 cells were also injected in the lateral tail veins of NSG and Balb/C mice, respectively to assess for colonization (metastasis) to other organs. Loss of WAVE2 expression resulted in a significant reduction in the number of lung metastases in mice injected with WAVE2-deficient MDA-MB-231 cells (Fig. 3D and E) and 4T1 (Fig. 3F and G). Metastatic foci were also observed in the liver from mice injected with CTRL MDA-MB-231 cells, but not with their W2KO derivatives (Fig. 3D). Therefore, our *in vivo* findings confirm that the loss of WAVE2 inhibits the rate of primary tumor growth and metastasis in both human and mouse models for TNBC.

### Loss of WAVE2 Inhibits *ITGB1* Expression Which Negatively Affects Interaction of Cancer Cells with the ECM

To investigate the molecular mechanisms underlying the role of WAVE2 in mediating TNBC tumor growth and metastasis, we generated RNA-seq from control and W2KO MDA-MB-231 (Supplementary Data S1) and MDA-MB-468 cells (Supplementary Data S2). Pathway analysis of the RNA-seq data identified the ECM interaction pathway among the most affected pathway as the result of loss of WAVE2. (Fig. 4A; Supplementary Fig. S5). Subsequent analyses revealed *ITGB1* and ITGA6 integrins, which are part of the ECM interaction pathway, to be significantly inhibited in the W2KO cells (Fig. 4B and C). Interrogation of the cBioPortal breast cancer datasets confirmed the positive correlation in expression levels between WAVE2 and *ITGB1* in human breast cancer specimens (Fig. 4D). Expression levels of *ITGB1*, similar to those of WAVE2, were also found to be elevated in the more aggressive basal breast cancer cell lines (Supplementary Fig. S6A). We also confirmed inhibition of expression of *ITGB1* in the W2KO MDA-MB-231 and 4T1 cells at the protein levels by immunoblotting (Fig. 4E), by IF (Fig. 4F), and by flow cytometry analyses to show loss of cell surface expression (Fig. 4G) and activity (HUTS4 binding assay) of *ITGB1* (Fig. 4H). In all cases, we used ITGB1KO as a control.

**FIGURE 5:**
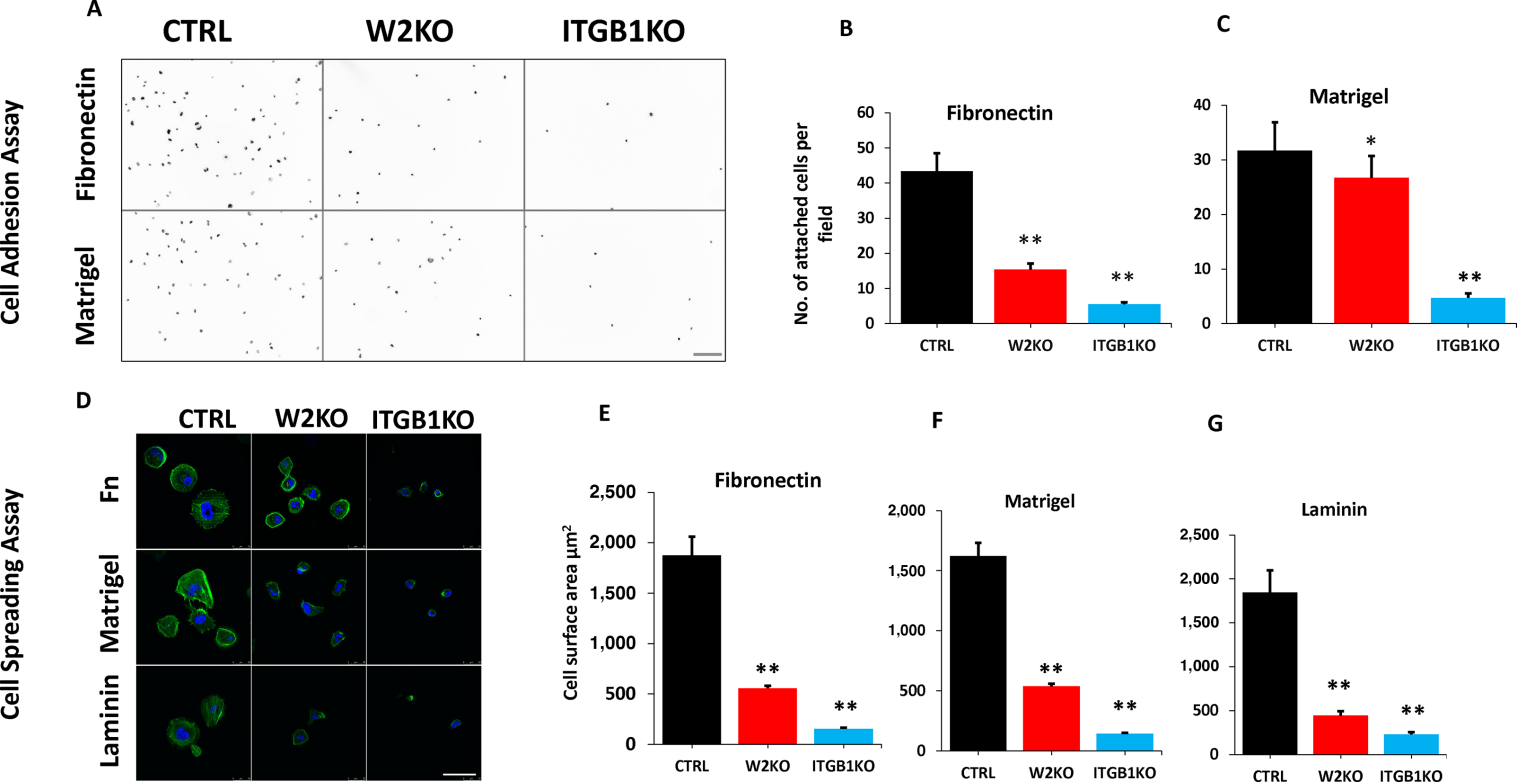
Loss of WAVE2 inhibits the *ITGB1*-mediated interaction of cancer cells with the extracellular matrix. Representative microscopic images of nuclei of CTRL, W2-KO, and *ITGB1*-KO MDA-MB-231 cells seeded on fibronectin- (top) or Matrigel-coated coverslips (bottom), and allowed to adhere for 30 minutes. Scale bar: 250 μm. Quantification of adhered cells on fibronectin (**B**) and Matrigel (**C**). Each dot corresponds to the nucleus of an adherent cell. **D,** Confocal microscopy images of IF staining of CTRL, W2-KO and *ITGB1*-KO MDA-MB-231 cells seeded on fibronectin- (top), Matrigel- (middle), or Laminin-coated coverslips (bottom), and allowed to spread overnight and stained for actin (green). Nuclei were counterstained with DAPI. Scale bar: 50 μm. Quantification of cell adhesion by means of cell surface area on fibronectin (**E**), Matrigel (**F**), and laminin (**G**). Data are the means ± SD (*n*  =  3; **, *P* < 0.01; Student *t* test). Data shown are representative of three replicates.

By interacting with ECM, integrins are critical regulators of cell adhesion and spreading, which are major hallmarks of cancer cell migration and invasion (14). Accordingly, we assessed the effect of loss of WAVE2 on *ITGB1*–mediated regulation of cell adhesion and spreading. Loss of WAVE2 (W2KO) inhibited the ability of MDA-MB-231 cells to adhere to fibronectin-coated culture plates to levels close to those achieved by ITGB1KO (Fig. 5A, top and 5B). Of note, *ITGB1* is a major receptor of the ECM fibronectin. W2KO also inhibited adhesion to the more generic ECM mix (Matrigel), albeit to levels that are less pronounced to those achieved by ITGB1KO (Fig. 5A, bottom and 5C). Loss of WAVE2 expression also inhibited cell spreading of MDA-MB-231 cells on fibronectin (Fig. 5D, top and 5E), Matrigel (Fig. 5D, middle and 5G), and laminin (Fig. 5D, bottom and 5H). Inhibition of cell spreading was almost similar between W2KO and ITGB1KO with all three ECM substrata. Thus, we show that loss of WAVE2 expression, by inhibiting *ITGB1* expression, negatively affects integrin-mediated regulation of major cell–ECM interactions, as determined by loss of cell adhesion and spreading to ECM. To further confirm that the observed phenotypes (cell adhesion and spreading) were indeed the result of inhibition of *ITGB1*, we assessed for the Src and FAK signaling activities, which are established down effectors of *ITGB1*, and found inhibition of *ITGB1* both in ITGB1KO or W2KO cells to inhibit phosphorylation of both Src and FAK (Supplementary Fig. S6B and S6D), therefore confirming our findings.

### The WAVE2 Modulation of Integrin Activities is Mediated Through the Regulation of miR-29 microRNA

**FIGURE 6:**
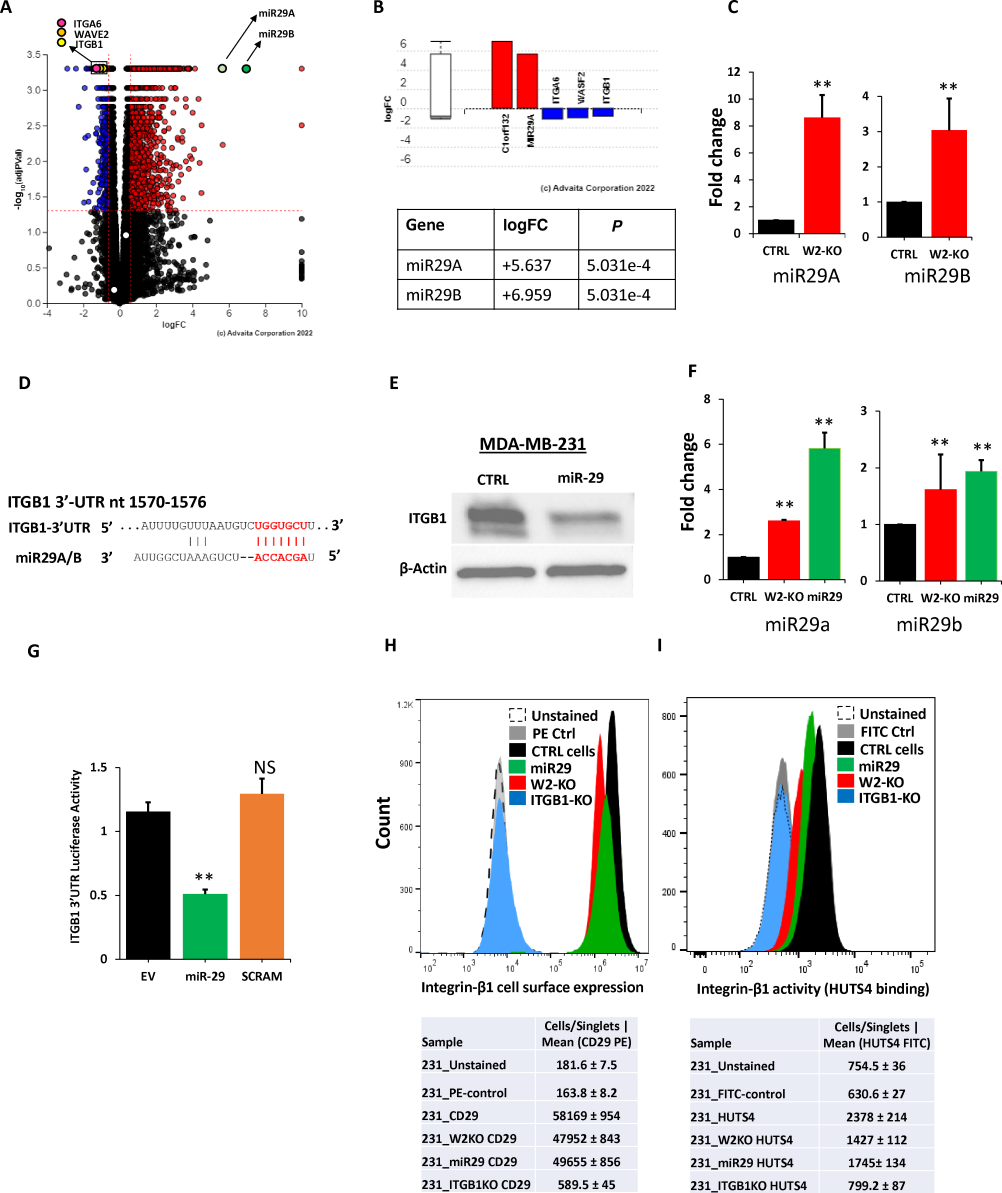
The WAVE2 modulation of integrin activities is mediated through the regulation of miR-29 miRNA. **A,** Volcano plot from the RNA-seq analysis of the differentially expressed genes between CTRL and W2-KO MDA-MB-231 cells. The *X*-axis represents the log_2_ fold change in expression levels and the *Y*-axis shows the *P* values. Red dots: upregulated genes; Blue dots: downregulated genes; Black dots: no significant change. The dots of the genes of interest are labeled. **B,** Bars and values representation of the log_2_ fold change in expression levels of miR-29a and miR-29b derived from the RNA-seq data. **C,** Quantification of miR-29a (left) and miR-29b (right) expression levels from CTRL and W2KO MDA-MB-231 cells using qRT-PCR. Values are plotted as fold change to the CTRL cells, after normalization to U6 expression levels. **D,** Nucleotide sequence and location of the seed sequence of miR-29 in the 3′-UTR of *ITGB1* mRNA. The sequence alignment with the miR-29 sequence is also shown. **E,** Representative WB analysis of protein lysates from CTRL and miR-29–expressing MDA-MB-231 probed with anti *ITGB1* antibody. β-Actin was used for loading control. **F,** Quantification of miR-29a (left) and miR-29b (right) expression levels from CTRL, W2KO, or miR-29–expressing MDA-MB-231 cells using qRT-PCR. Values are plotted as fold change to the CTRL cells, after normalization to U6 expression levels. **G,** Quantification of luciferase activity of *ITGB1*–3′-UTR. Firefly luciferase reporter plasmid pmirGlo, empty (EV), containing the 3′-UTR of *ITGB1* with wild-type (miR-29) or scrambled miR-29 seed sequence (SCRAM) was transiently transfected into HEK293 along with a miR-29–expressing vector. Luciferase activities were measured after 48 hours and plotted after being normalized *Renilla* luciferase. Representative histograms of flow cytometry analyses for cell surface expression of *ITGB1* (**H**) and activity: HUTS4 binding (**I**) of CTRL, W2-KO, and *ITGB1*-KO or miR-29–expressing MDA-MB-231 cells. Shift to the right indicates increased expression or activity. Data are the means ± SD (*n*  =  3; **, *P* < 0.01; Student *t* test). Data shown are representative of three replicates.

To further investigate the mechanisms whereby WAVE2 regulates *ITGB1* and its downstream signaling, we went back to our RNA-seq data and found expression levels of both miR-29a, as well as miR-29b to be significantly (*P* < 0.001) higher in the W2KO MDA-MB-231 cells compared with their control counterparts (Fig. 6A and B). We independently confirmed this finding by qRT-PCR (Fig. 6C). miRNAs are small single-stranded noncoding RNAs that function in posttranslational regulation of gene expression by directly binding to the 3′-UTR of the target gene. Having hypothesized that miR-29 might regulate expression of *ITGB1*, we surveyed the 3′-UTR of *ITGB1* and found a conserved target site of miR-29 (Fig. 6D; Supplementary Fig. S7A and S7B). Indeed, overexpression of miR-29 in MDA-MB-231 cells inhibited expression of *ITGB1* (Fig. 6E), further supporting our hypothesis. Overexpression of both miR-29a and miR-29b were confirmed by qRT-PCR (Fig. 6F). To further demonstrate that the posttranscriptional repression of the *ITGB1* transcript is caused by direct binding of miR-29 to its target seed sequence within the 3′-UTR of *ITGB1*, we used the Firefly-Renilla dual luciferase reporter gene assay. We subcloned a part of *ITGB1* 3′-UTR that contains the miR-29 binding sequence in the pmirGlo vector, and luciferase activity was measured in HEK293 cells transfected with the pmirGlo-*ITGB1*-3′-UTR along with miR-29–expresing plasmid or its empty control vector. Overexpression of miR-29 in HEK cells transfected with the pmirGlo-*ITGB1*-3′-UTR resulted in approximately 60% reduction of luciferase activity, compared with cell transfected with empty vector (Fig. 6G). As an independent approach to confirm that miR-29 specifically targets and binds to its seed sequence in the 3′-UTR of *ITGB1* to reduce its expression, we scrambled the seed sequence of miR-29 target in the 3′-UTR of *ITGB1*. This manipulation abrogated the effect of exogenous miR-29 in HEK cells (Fig. 6G). Overexpression of miR-29 in MDA-MB-231 cells also inhibited cell surface (Fig. 6H) and activation (Fig. 6I) levels of *ITGB1*, as assessed by FACS and HUTS-4 binding assays. Thus, we confirmed that expression of *ITGB1* is regulated by miR-29 downstream of WAVE2.

**FIGURE 7:**
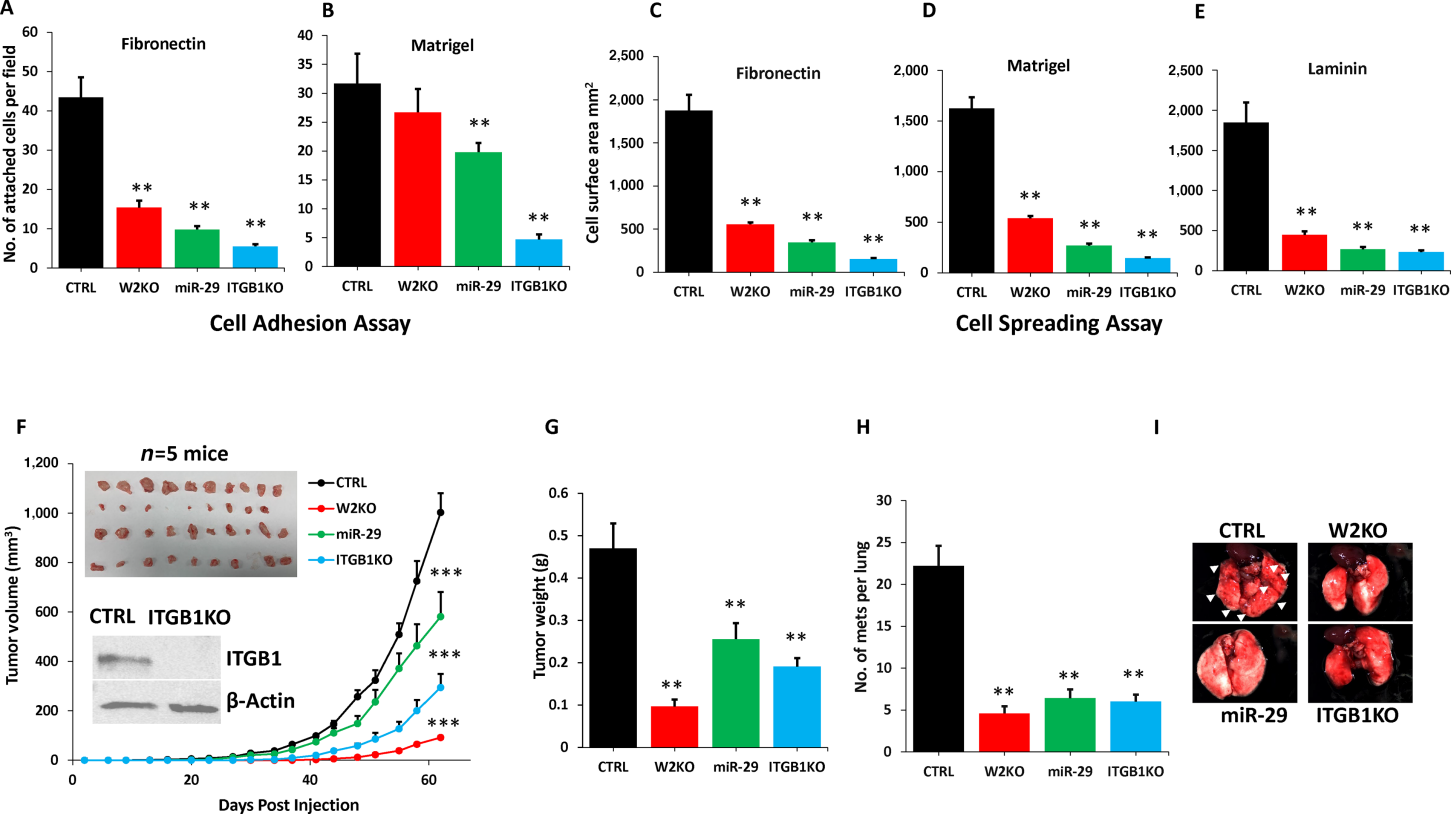
The WAVE2-mediated modulation of miR-29 expression regulates of *ITGB1* oncogenic activities. Quantification of cell adhesion of CTRL, W2KO, *ITGB1*-KO, or mi-R29–expressing MDA-MB-231 cells on fibronectin (**A**) and Matrigel (**B**). Quantification of cell adhesion of CTRL, W2KO, *ITGB1*-KO, or miR-29–expressing MDA-MB-231 cells on fibronectin (**C**), Matrigel (**D**), and laminin (**E**). Data are the means ± SD (*n*  =  3; **, *P* < 0.01; Student *t* test). Data shown are representative of three replicates. **F,** Quantification of volume of tumors derived from implantation of CTRL, WAVE2-KO, *ITGB1*-KO, or miR-29–expressing MDA-MB-231 cells into the mammary fat pads of NSG mice. The inserts show WBs confirming *ITGB1*-KO in MDA-MB-231 cells before mice injections, as well images of the resulting tumors from each group. **G,** Quantification of tumor weights. **H,** Quantification of lung metastatic foci. **I,** Images of lungs from the corresponding experiment. Arrow heads point metastatic foci that are abundant in the lungs of CTRL mice compared their W2KO, miR-29, and ITGB1KO counterparts. **, ***, *P* < 0.01; Student *t* test.

To further assess the effect of the miR-29–mediated downregulation of *ITGB1* we subjected MDA-MB-231 CTRL cells and their W2KO, miR-29–overexpressing and ITGB1KO derivatives to cell adhesion and spreading assays, and found that the miR-29–expressing cells show reduced cell adhesion on Fibronectin (Fig. 7A) and Matrigel (Fig. 7B), and reduced cell spreading on Fibronectin (Fig. 7C), Matrigel (Fig. 7D), and Laminin (Fig. 7E). ITGB1KO cells served as a negative control in these experiments. Next, to investigate the effect of miR-29 overexpression on tumor growth and metastasis, we injected mammary fat pads of 6–8 weeks old NSG mice with MDA-MB-231 CTRL cells or their W2KO, miR-29–overexpressing or ITGB1KO derivatives, and tumor growth was assessed over 8 weeks. We found overexpression of miR-29 to significantly inhibit tumor growth in the same manner that W2KO or ITGB1KO did (Fig. 7F and G). Lung metastasis was also inhibited as a result of miR-29 overexpression. (Fig. 7H and I). Together, these finding confirm the hypothesis that miR-29, when upregulated in the W2KO cells, downregulates *ITGB1* by directly binding to its 3′-UTR, which in turn causes the cancer cells to lose their oncogenic properties *in vitro* and their tumor growth and metastasis promoting potentials *in vivo*.

### The miR-29 Targets WAVE2 in a Negative Feedback Loop to Modulate Expression and Activity of *ITGB1*

**FIGURE 8:**
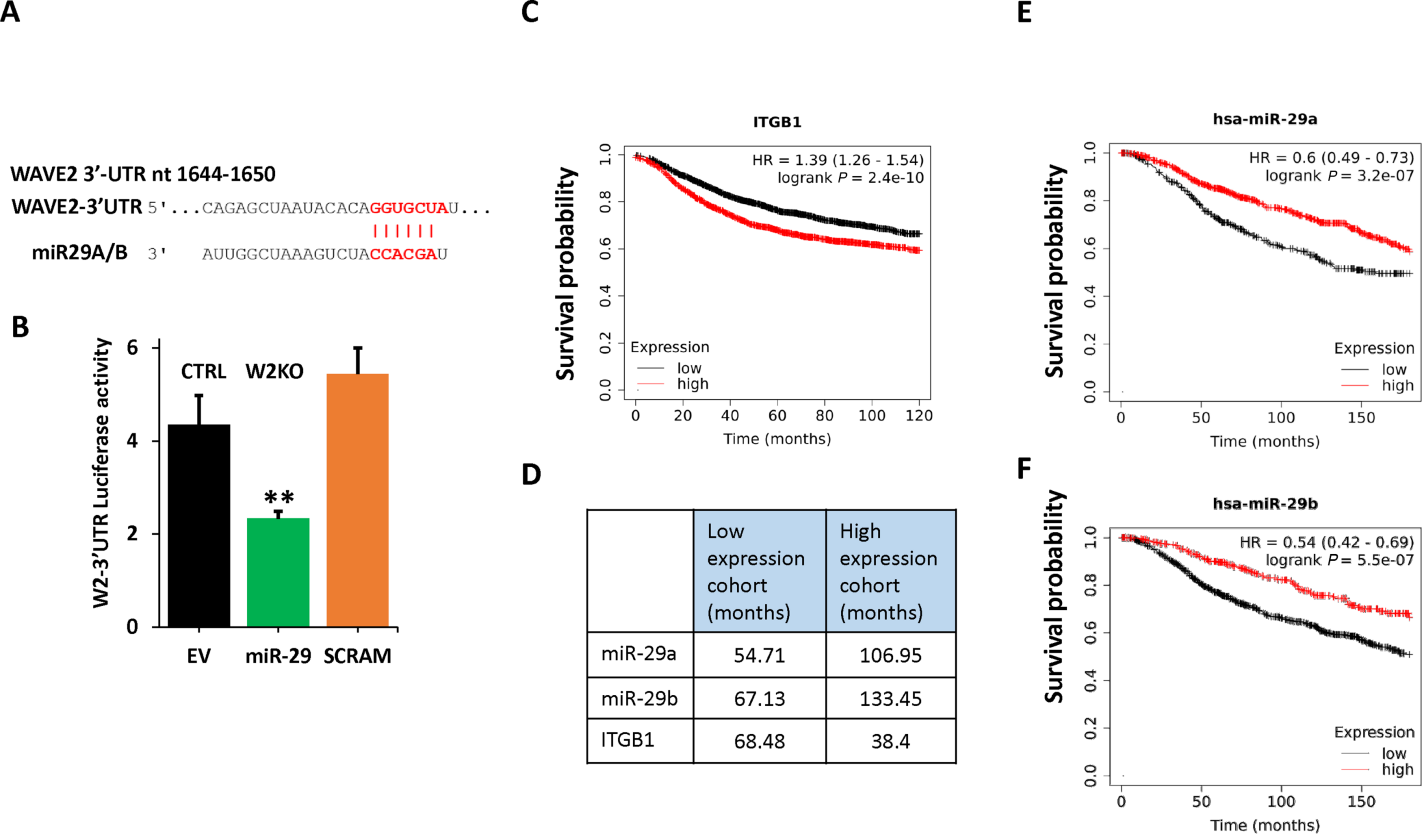
The miR-29 targets WAVE2 in a negative feedback loop to modulate expression and activity of *ITGB1*. **A,** Nucleotide sequence and location of the seed sequence of miR-29 in the 3′-UTR of WAVE2 mRNA. The sequence alignment with the miR-29 sequence is also shown. **B,** Quantification of luciferase activity of W2–3′-UTR. Firefly luciferase reporter plasmid pmirGlo, empty (EV), containing the 3′-UTR of WAVE2 with wild-type (miR-29) or scrambled miR-29 seed sequence (SCRAM) was transiently transfected into HEK293 along with a miR-29–expressing vector. Luciferase activities were measured after 48 hours and plotted after being normalized *Renilla* luciferase. **, *P* < 0.01; Student *t* test. Data shown are representative of three replicates. KM plot correlating survival of 4.929 patients with breast cancer with mRNA expression levels of *ITGB1* (**C**), miR-29a (**D**), and miR-29b (**E**). Low expression levels of miR-29a or miR-29b correlate with poor survival probability in patients with breast cancer. Number of patients at risk and median survival in the low and high WAVE2 cohorts are shown in **F**.

The data presented in Fig. 6 show that loss of WAVE2 expression results in increased expression of miR-29, which in turn inhibits *ITGB1* expression and its downstream signaling. Interestingly, a survey of the WAVE2 3′-UTR identified a conserved target site for miR-29 (Fig. 8A; Supplementary Fig. S8A and S8B). We confirmed the specific binding of miR-29 to its target in the WAVE2-3′-UTR using Firefly-Renilla dual luciferase reporter gene assay; miR-29 inhibited luciferase activity in the presence of wildtype WAVE2-3′-UTR, but not in the presence of the mutated (SCRAM) miR-29 target site (Fig. 8B). These new findings identified a possible negative feedback loop between WAVE2 and miR-29, that coalesces to the regulation of *ITGB1*. Interrogation of the breast cancer KM plotter datasets showed that, in concordance with WAVE2 (Fig. 1G), increased levels of *ITGB1* correlate with decreased survival probability of patients with breast cancer (Fig. 8C and D), while increased expression levels of miR-29a (Fig. 8D and E) and miR-29b (Fig. 8D and F) have the opposite effect. Together, our findings support the interrelationship between WAVE2 and miR-29 in the regulation of the *ITGB1*–mediated promotion of breast cancer tumor growth and metastasis, through the negative regulatory feedback loop between WAVE2 and miR-29.

### Reexpression of Exogenous WAVE2 in the WAVE2-deficient TNBC Cells Restores *ITGB1* Expression and Activity

**FIGURE 9:**
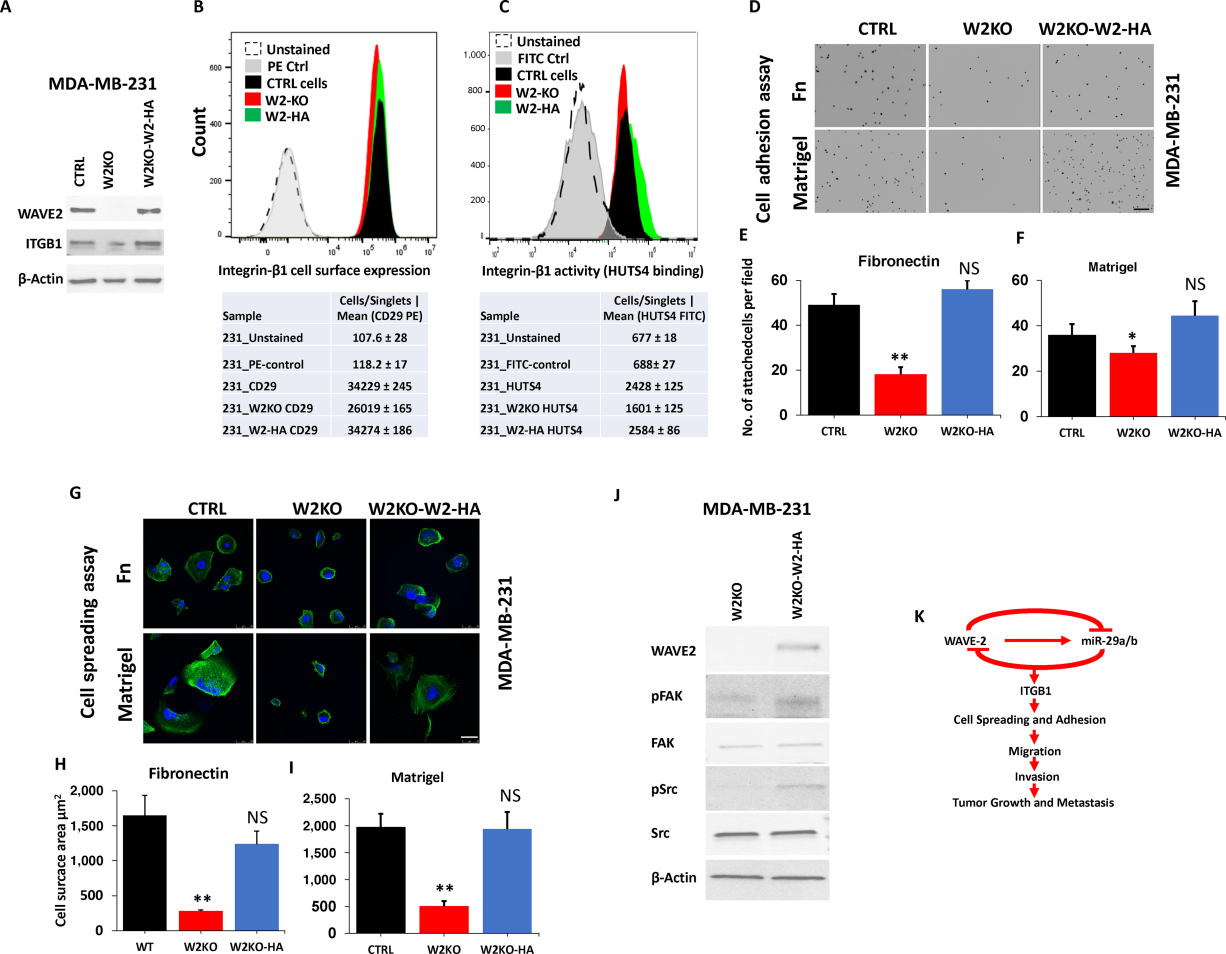
Reexpression of Exogenous WAVE2 in the WAVE2-deficient TNBC cells restores *ITGB1* expression and activity. **A,** Representative WB analysis of protein lysates from CTRL, W2-KO, and W2-KO MDA-MB-231 overexpressing HA-tagged WAVE2 probed with the indicated antibodies. β-Actin was used for loading control. Representative histograms of flow cytometry analyses for cell surface expression of *ITGB1* (**B**) and activity: HUTS4 binding (**C**) of CTRL MDA-MB-231, W2-KO, and W2-KO cells overexpressing HA-tagged WAVE2. Shift to the right indicates increased expression or activity. Data are the means ± SD (*n*  =  3; **, *P* < 0.01; Student *t* test). Data shown are representative of three replicates. **D,** Representative confocal microscopy images of nuclei of CTRL, W2-KO, and W2-KO MDA-MB-231 overexpressing HA-tagged WAVE2 seeded on fibronectin- (top) or Matrigel-coated coverslips (bottom), and allowed to adhere for 30 minutes. Scale bar: 250 μm. **E** and **F,** Quantification of adhered cells on fibronectin (**B**) and Matrigel (**C**). Each dot corresponds to the nucleus of an adherent cell. **G,** Confocal microscopy images of IF staining of CTRL, W2-KO, and W2-KO MDA-MB-231 overexpressing HA-tagged WAVE2 seeded on fibronectin (top) or Matrigel (bottom), and allowed to spread overnight and stained for actin (green). Nuclei were counterstained with DAPI. Scale bar: 50 μm. Quantification of cell adhesion by means of cell surface area on fibronectin (**H**), and Matrigel (**I**). Data are the means ± SD (*n*  =  3; **, *P* < 0.01; Student *t* test). Data shown are representative of three replicates. **J,** Representative WB analysis of protein lysates from W2-KO and W2-KO MDA-MB-MB-231 overexpressing HA-tagged WAVE2 probed with the indicated antibodies. β-Actin was used for loading control. **K,** A diagram depicting the negative feedback signaling loop between WAVE2 and miR-29b, and the consequences of its regulatory effect on *ITGB1* on the invasion-metastasis cascade of breast cancer tumors.

To confirm the specificity of loss of WAVE2 on *ITGB1* expression and activity, as well as the downstream phenotypic and signaling affects, we used an HA-tagged WAVE2-expressing plasmid to restore WAVE2 expression. First, we transiently expressed HEK-293 cells with either the empty vector or the HA-WAVE2–expressing vector, and confirmed expression of exogenous WAVE2 by WB analyses using anti-HA antibody to detected HA-WAVE2 fusion exogenous WAVE2, and anti-WAVE2 antibody to detect both endogenous and exogenous WAVE2 (Supplementary Fig. S9). Next, we overexpressed HA-WAVE2 in W2-KO-MDA231 cells and showed an increase in *ITGB1* signal compared with the W2-KO cells, as well as a slight increase, compared with the control MDA-231 cells (Fig. 9A). We also used flow cytometry analyses to confirm that overexpression of WAVE2 in the W2-deficient MDA-MB-231 cells also resulted in increased *ITGB1* cell surface expression (CD29; Fig. 9B) and activity (HUTS4; Fig. 9C). Reexpression of WAVE2 in the W2-deficient MDA-MB-231 cells also restored the ability of these cells to adhere to Fibronectin (Fig. 9D and E) and to Matrigel (Fig. 9D and F). Cell spreading was also restored on the same ECM substrata (Fig. 9G and H, for Fibronectin and Fig. 9G and I, for Matrigel), as a result of rescue of WAVE2 expression. Finally, to further confirm that the observed phenotypes (cell adhesion and spreading) were indeed the result of the rescue of *ITGB1* activity, we assessed for the downstream effectors of *ITGB1*, Src and FAK, and found reexpression of WAVE2 restored phosphorylation of both Src and FAK (Fig. 9J), therefore confirming the specificity of WAVE2 in regulating *ITGB1* expression and activity.

## Discussion

TNBC is one of the most challenging subtypes of breast cancers to treat and has accounted for poor survival rates in diagnosed patients due to its invasive nature. A myriad of signaling reactions have been investigated that are responsible for tumor growth, invasiveness, and metastasis. Several oncogenes have been documented to orchestrate these signaling pathways to promote cancer. Among them a member of Wiskott–Aldrich syndrome protein family, WAVE2, has recently drawn attention toward its role in cancer progression and metastasis. However, the extent to which WAVE2 regulates tumorigenesis and metastasis and the molecular mechanisms by which it subserves such roles remain largely unknown. Because metastasis is the major cause of death in patients with breast cancer, it becomes critical to investigate the molecular mechanisms whereby certain factors such as actin cytoskeleton remodeling proteins (WAVE2), regulators of cell adhesion and spreading (integrins), and regulators of global posttranscriptional repression (miRNAs), and how interrelationships between these molecular mechanisms impact cancer progression and metastasis, which may provide novel therapeutic alternatives and opportunities to treat breast cancer. In the current study, we used a combination of global transcriptomics (RNA-seq), bioinformatics, genetic manipulation of gene expression, different biochemical and cell imaging analyses in vitro, in addition to *in vivo* mouse models for TNBC, as well as interrogation of public human breast cancer datasets, to investigate the potential role of WAVE2 and its downstream effectors (miR-29 and *ITGB1*) in TNBC tumor progression and metastasis. As such, we identified a novel WAVE2/miR-29/*ITGB1* signaling axis (Fig. 9K) that regulates TNBC tumor growth and metastasis. More importantly, we identified a negative regulatory feedback loop between WAVE2 and miR-29, where inhibition of WAVE2 expression activates miR-29. In turn, miR-29 feeds back and targets WAVE2 to repress its expression. In addition to WAVE2, miR-29 targets and represses expression of *ITGB1*. In fact, analysis or our RNA-seq data showed that loss of WAVE2 expression results in the repression of not only *ITGB1*, but several other integrins, including ITGA6, ITGAV, ITGA2, ITGB4, and ITGB5 (Supplementary Data S1 and S2; Supplementary Fig. S10). This study focused mainly on *ITGB1*, given its established role in the regulation of several major hallmarks of cancer (35). Our future studies will investigate the novel role of miR-29 as a global regulator of integrins in cancer.

Our data established a negative feedback loop between WAVE2 and miR-29. While the miR-29–mediated regulation of WAVE2 was determined to be through the binding of miR-29 to its target site in the WAVE2 3′-UTR, the mechanism whereby WAVE2 regulates miR-29 was not definitely established. However, while our data explain how miR-29 might regulate WAVE2 (posttranslational regulation), it does not explain how WAVE2 regulates miR-29 expression. A potential mechanism was revealed from our RNA-seq data, where we found loss of WAVE2 expression results in increased expression levels of DGCR8 mRNA (Supplementary Fig. S11A and S11B). DGCR8, also known as PASHA, is an important component of the miRNA biogenesis machinery, where DGCR8, along with DROSHA, are required for the processing of pri-miRNA to the pre-miRNA form before it is exported from nucleus to the cytoplasm to be incorporated in the RISC complex. WB analyses confirmed increased expression levels of DGCR8 protein in both the W2KO and the miR-29–overexpressing MDA-MB-231 cells (Supplementary Fig. S11C and S11D). On the other hand, Supplementary Fig. S11E shows an inverse correlation in expression levels between WAVE2 and DGCR8 in breast cancer cell lines, while interrogation of the KP plotter breast cancer datasets confirmed the positive correlation between increased DGCR8 expression levels and survival probability of human patients with breast cancer tumors (Supplementary Fig. S11F). Therefore, we identified one plausible mechanism, whereby WAVE2 may regulate DGCR8 expression: downregulation of WAVE2 resulted in increased expression of DGCR8, which in turn increase the availability of pre-miR-29 miRNA pool to be exported to the cytoplasm and further processed by DICER, before it is incorporated in the RISC complex. Given the role of DGCR8 as a global regulator of maturation of miRNAs inside the nucleus, we are cognizant that the WAVE2-mediated stabilization of DGCR8 may not be the only mechanism whereby miR-29 expression is regulated in our system, and, therefore, more in-depth investigation is required. WAVE2 is known to play a critical role in cytoskeletal actin reorganization (11). The spatial reorganization of actin cytoskeleton is crucial for maintaining cellular morphology and functions (12). Cytoskeletal actin dynamics mediate the regulation of transcriptional machinery in the nucleus which is crucial in RNA processing (36, 37). Any fluctuations in WAVE2 expression levels may cause changes in actin cytoskeleton reorganization, which could in turn affect the global transcriptomic machinery. This could provide another way to explain how changes in WAVE2 expression affect the observed changes in miR-29 expression. Another mechanism whereby WAVE2 may regulate DGCR8 expression could be through its translocation to the nucleus, where it may bind to DCGR8 and stabilize it, or through its inclusion in the transcription machinery by acting as a transcription factor or biding and activating DCGR8-specific transcription factors. To support this theory, we show localization of WAVE2 in the nucleus via IF (Supplementary Fig. S12A) and WB (Supplementary Fig. S12B). All these plausible mechanisms need, however, to be investigated.

All three WAVE isoforms (1, 2, and 3) have been heavily investigated for their original description as major regulators of actin cytoskeleton (38). For the past 20 years, our group has been investigating the role of WAVE3 in tumor progression and metastasis (6, 34), and have clearly shown that neither WAVE1 nor WAVE2 can compensate for loss of WAVE3. In this study, we show again that loss of WAVE2 in breast cancer cell lines and tumors can not be compensated for by neither WAVE1 nor WAVE3, even though their expression levels remain unchanged in the WAVE2-KO cells. Loss of WAVE2 had a dramatic effect on lamellipodia at the leading edge of MDA-MB-231 cells (Supplementary Fig. S13), in a similar manner that did loss of WAVE3 (39). However, the signaling mechanisms by which they regulate actin cytoskeleton dynamics must be specific to each isoform, because loss of one isoform cannot be compensated for by a different isoform. The literature also provides evidence in support of the specific functions of the different WAVE isoforms (13, 40); global deletion of either WAVE1 (41) or WAVE2 (42) in mice results in embryonic lethality or premature death just a few days after birth, which support the notion that the function of any given WAVE isoform cannot be compensated for by the other isoforms even when they are coexpressed, further supporting the temporal and special specificity of function of each WAVE.

Overall, our findings suggest that combining miR-29 protagonists, combined with inhibition of WAVE2 and/or integrins may provide synergistic benefits in mitigating the invasive and metastatic properties of TNBC tumors. Future studies will explore the utility of combined miRNA therapy targeted against WAVE2 as a better therapeutic alternative for chemotherapies for the treatment of TNBC tumors.

## Supplementary Material

Supplementary Table ST1 Supplementary Table containing primer sequences

Supplementary Figure S1 Relationship between WAVE2 expression levels and breast Cancer subtypes and disease outcome.

Supplementary Figure S2 Effect of loss of WAVE2 expression on tumorsphere invasion.

Supplementary Figure S3 Hair plot of volumes of individual tumors of derived from control and WAVE2-KO MDA-231 or 4T1 BC cell lines.

Supplementary Figure S4 Quantification of volume of tumors derived from Control or WAVE2-KO MDA-MB-468 cells.

Supplementary Figure S5 Pathway analysis of RNA-seq data generated from CTRL and WAVE2-KO MDA-MB-231 cells.

Supplementary Figure S6 Western Blot analyses of WAVE2 and ITGB1 expression in different BC cell lines, and effect of loss of WAVE2 expression on levels of ITGB1, pSRC and pFAK.

Supplementary Figure S7 Target Scan prediction of micoRNA target sites in the 3'UTR of ITGB1.

Supplementary Figure S8 Target Scan prediction of micoRNA target sites in the 3'UTR of WAVE2.

Supplementary Figure S9 Western Blot analyses confirming exogenous expression of HA-tagged WAVE2 in HEK-293 cells.

Supplementary Figure S10 Volcano plot from the RNA-seq analysis of the differentially expressed genes between CTRL and W2-KO MDA-MB-231 cells.

Supplementary Figure S11 Analysis of the potential role of DGCR8 in the WAVE2-mediated regulation of miR-29 in BC.

Supplementary Figure S12 Immunofluorescence and Western Blot analyses of WAVE2 distribution between the cytoplasmic and the nuclear compartments of MDA-MB-231 cells.

Supplementary Figure S13 Immunofluorescence analysis of the effect of loss of WAVE2 on lamellipodia formation in MDA-MB-231 cells.

Supplementary Data File SD1 Raw RNA-seq Data of control vs W2-KO MDA-MB-231 cells

Supplementary Data File SD2 Raw RNA-seq Data of Control vs W2KO-MDA-MB-468 cells
