# Supplementary material for: The WAVE2/miR-29/Integrin-β1 Oncogenic Signaling Axis Promotes Tumor Growth and Metastasis in Triple-negative Breast Cancer
Source: Cancer Res Commun. 2023 Jan 31;3(1):160–74. doi: 10.1158/2767-9764.CRC-22-0249 (PMC10035451; doi:10.1158/2767-9764.CRC-22-0249)
Supplement: Supplementary Table ST1 — Supplementary Table containing primer sequences [file crc-22-0249-s01.pdf]

Supplementary Table 1: Primer sequences

Name	Sequence	Product Length (bp)
ITGA6-miR29-F	5’ ACCCCACAACCCAAAAGGTT 3’	769
ITGA6-miR29-R	5’ GGACACAAGTTCCCCAAGCA 3’	
ITGB1-miR29-F	5’ TATGAATTCACTTTACAAATTCAAGCCTTA 3’	410
ITGB1-miR29-R	5’ TGAGAATTCAACATACAGTGGTCTGTTATG 3’	
W2-miR29-F	5’ CAAAGAATTCTGCCAACTGTGGA 3’	420
W2-miR29-R	5’ GGAGGGAATTCTGAGGGCTTTGGC 3’	
ITGA6-miR29-SCRAM-F	5’ ACAGCAAACATTGGACGATTTGTTTTG 3’	NA
ITGA6-miR29-SCRAM-R	5’ CAAAACAAATCGTCCAATGTTTGCTGT 3’	
ITGB1-miR29-SCRAM-F	5’ GTTTAATGTCGTTCGGTTTCTGTCACC 3’	NA
ITGB1-miR29-SCRAM-R	5’ GGTGACAGAAACCGAACGACATTAAAC 3’	
W2-miR29-SCARM-F	5’ CTAATACACATGCGATGTTATTCAGAAA 3’	NA
W2-miR29-SCRAM-R	5’ TTTCTGAATAACATCGCATGTGTATTAG 3’	
pmirGlo-F	5’ GAAGCTGAGTTGGCTGCT 3’	NA
