# Supplementary material for: The WAVE2/miR-29/Integrin-β1 Oncogenic Signaling Axis Promotes Tumor Growth and Metastasis in Triple-negative Breast Cancer
Source: Cancer Res Commun. 2023 Jan 31;3(1):160–74. doi: 10.1158/2767-9764.CRC-22-0249 (PMC10035451; doi:10.1158/2767-9764.CRC-22-0249)
Supplement: Supplementary Figure S1 — Relationship between WAVE2 expression levels and breast Cancer subtypes and disease outcome. [file crc-22-0249-s02.pdf]

A

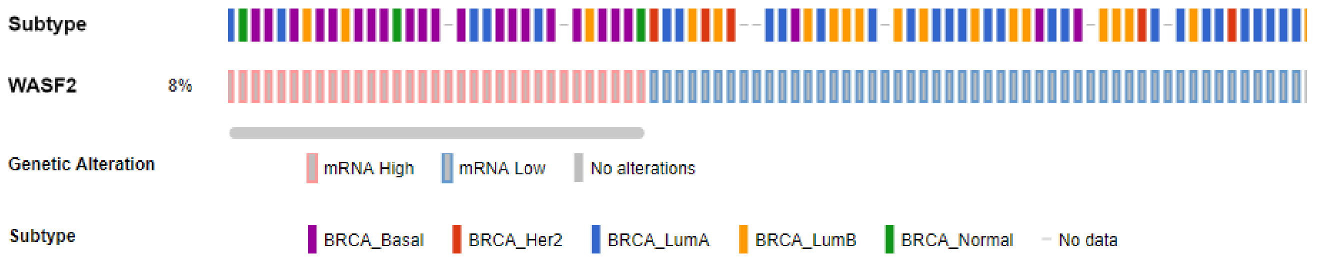

B

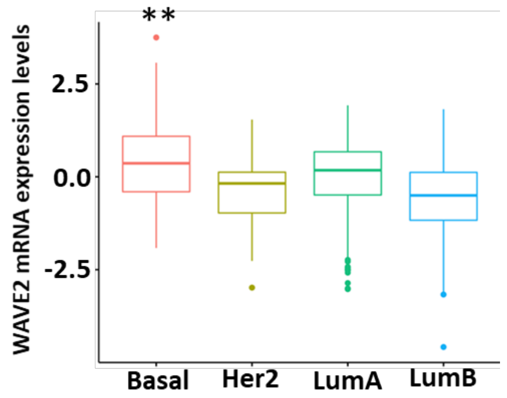

Sup. Fig. 1A&B. WAVE2 (WASF2) is upregulated in basal subtype of breast cancer. Data derived from the breast cancer datasets of the cBioPortal platform

C

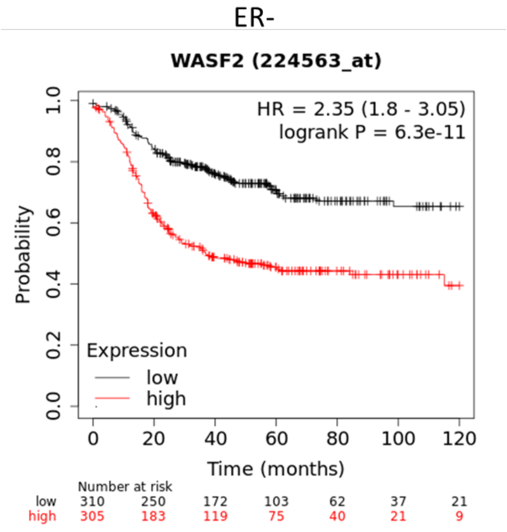

Low expression cohort (months)	High expression cohort (months)
43	14.64

D

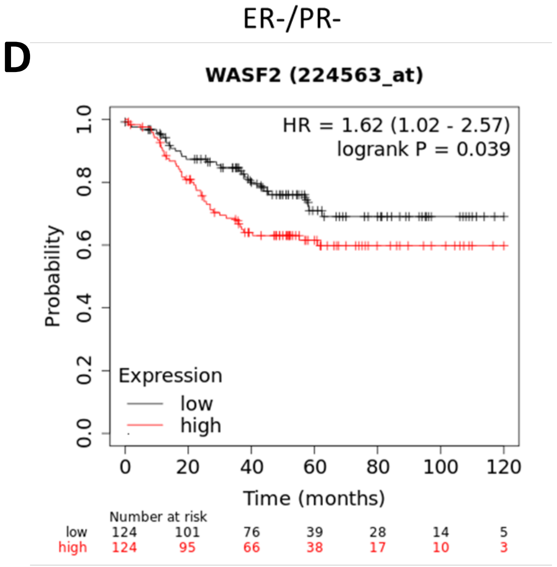

Low expression cohort (months)	High expression cohort (months)
57	25

Sup. Fig. 1C&D. increased expression levels of WAVE2 (WASF2) correlates with decreased survival probability in patients with ER-(C) and ER-/PR- BC tumors. Data derived from the BC KM-Plotter (https://kmplot.com/analysis)

Supplementary Figure S1
