# Supplementary material for: The WAVE2/miR-29/Integrin-β1 Oncogenic Signaling Axis Promotes Tumor Growth and Metastasis in Triple-negative Breast Cancer
Source: Cancer Res Commun. 2023 Jan 31;3(1):160–74. doi: 10.1158/2767-9764.CRC-22-0249 (PMC10035451; doi:10.1158/2767-9764.CRC-22-0249)
Supplement: Supplementary Figure S2 — Effect of loss of WAVE2 expression on tumorsphere invasion. [file crc-22-0249-s03.pdf]

Supplementary Figure S2

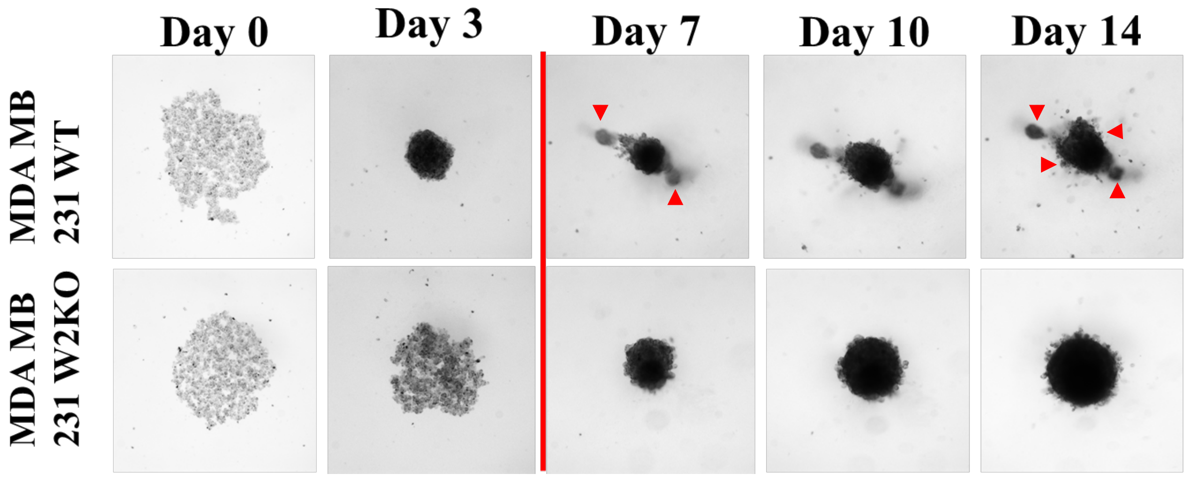

Sup. Fig.2. Representative micrographs of tumorspheres invasion from CTRL and W2KO MDA-MB-231. Tumorspheres were grown in a 96-well ULA plates and Matrigel (2.5 v/v) was added to the tumorsphere cultures at day 3 and images were captured using Incucyte. Red arrowheads point to invading microspheres.
