# Supplementary material for: The WAVE2/miR-29/Integrin-β1 Oncogenic Signaling Axis Promotes Tumor Growth and Metastasis in Triple-negative Breast Cancer
Source: Cancer Res Commun. 2023 Jan 31;3(1):160–74. doi: 10.1158/2767-9764.CRC-22-0249 (PMC10035451; doi:10.1158/2767-9764.CRC-22-0249)
Supplement: Supplementary Figure S3 — Hair plot of volumes of individual tumors of derived from control and WAVE2-KO MDA-231 or 4T1 BC cell lines. [file crc-22-0249-s04.pdf]

Supplementary Figure S3

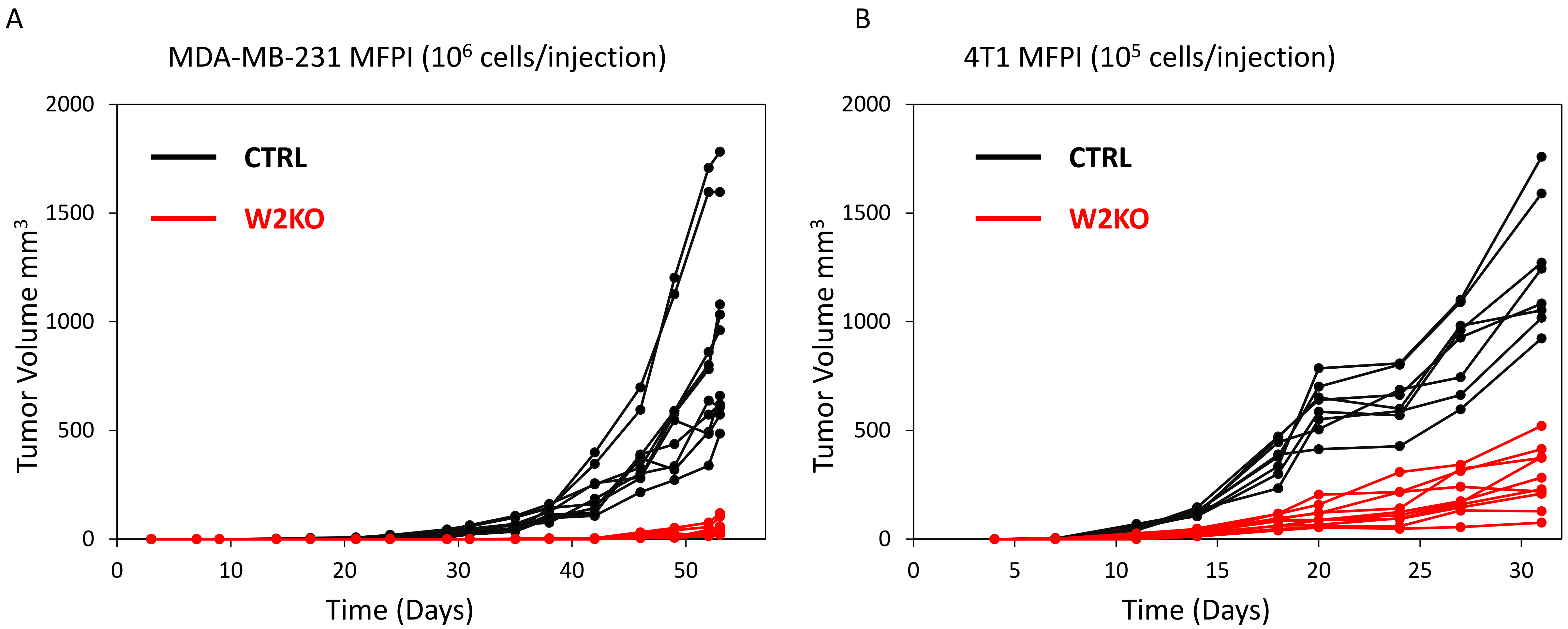

Sup. Fig. 3. Hair plot of volumes of individual tumors of CTRL or WAVE2-KO MDA-MB-231 (A) or 4T1 (B) implanted in the mammary fat pads of NSG or Balb/C mice
