# Supplementary material for: The WAVE2/miR-29/Integrin-β1 Oncogenic Signaling Axis Promotes Tumor Growth and Metastasis in Triple-negative Breast Cancer
Source: Cancer Res Commun. 2023 Jan 31;3(1):160–74. doi: 10.1158/2767-9764.CRC-22-0249 (PMC10035451; doi:10.1158/2767-9764.CRC-22-0249)
Supplement: Supplementary Figure S4 — Quantification of volume of tumors derived from Control or WAVE2-KO MDA-MB-468 cells. [file crc-22-0249-s05.pdf]

Supplementary Figure S4

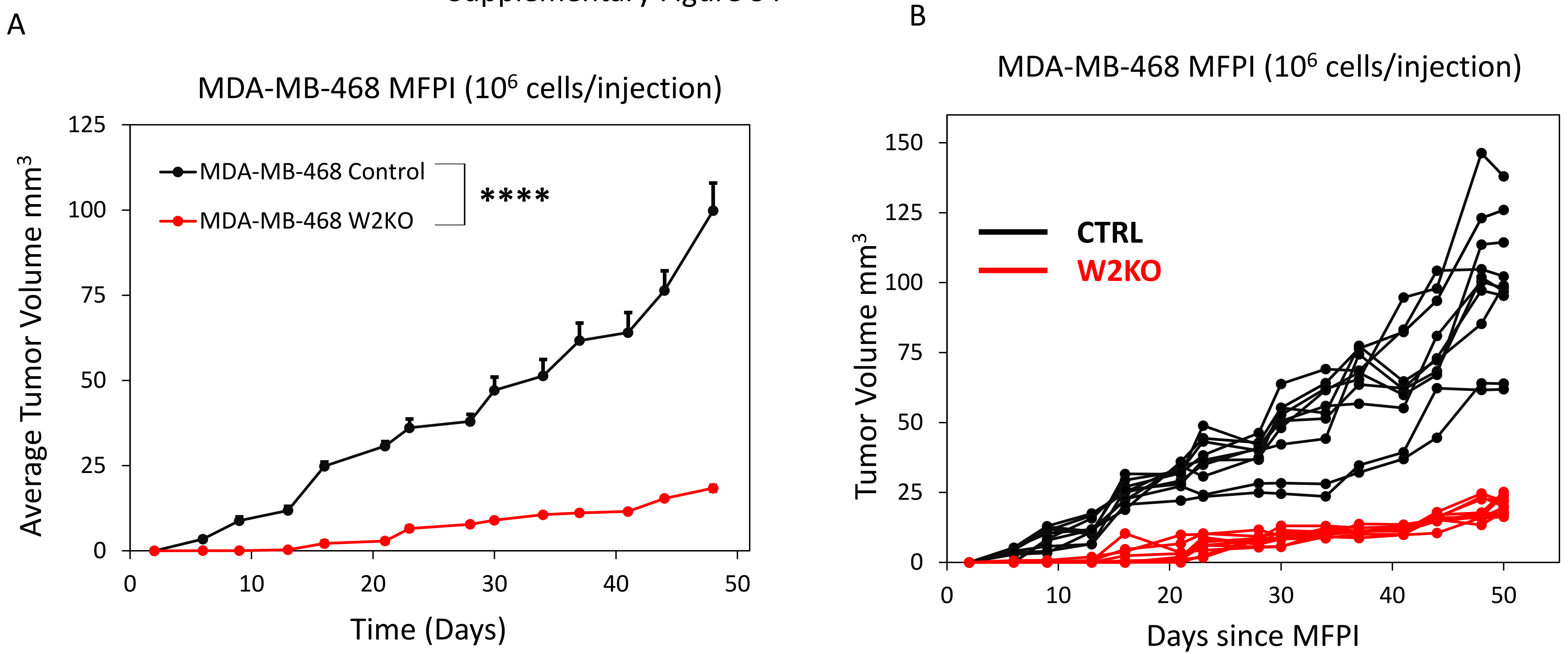

Sup Fig. 4. (A) Quantification of volume of tumors derived from implantation of CTRL or WAVE2-KO MDA-MB-468 cells into the mammary fat pads of NSG mice. ***, $p < 0.01$; Student's t-test. (B) Hai plots of volumes of individual tumors shown in (A).
