# Supplementary material for: The WAVE2/miR-29/Integrin-β1 Oncogenic Signaling Axis Promotes Tumor Growth and Metastasis in Triple-negative Breast Cancer
Source: Cancer Res Commun. 2023 Jan 31;3(1):160–74. doi: 10.1158/2767-9764.CRC-22-0249 (PMC10035451; doi:10.1158/2767-9764.CRC-22-0249)
Supplement: Supplementary Figure S5 — Pathway analysis of RNA-seq data generated from CTRL and WAVE2-KO MDA-MB-231 cells. [file crc-22-0249-s06.pdf]

Supplementary Figure S5

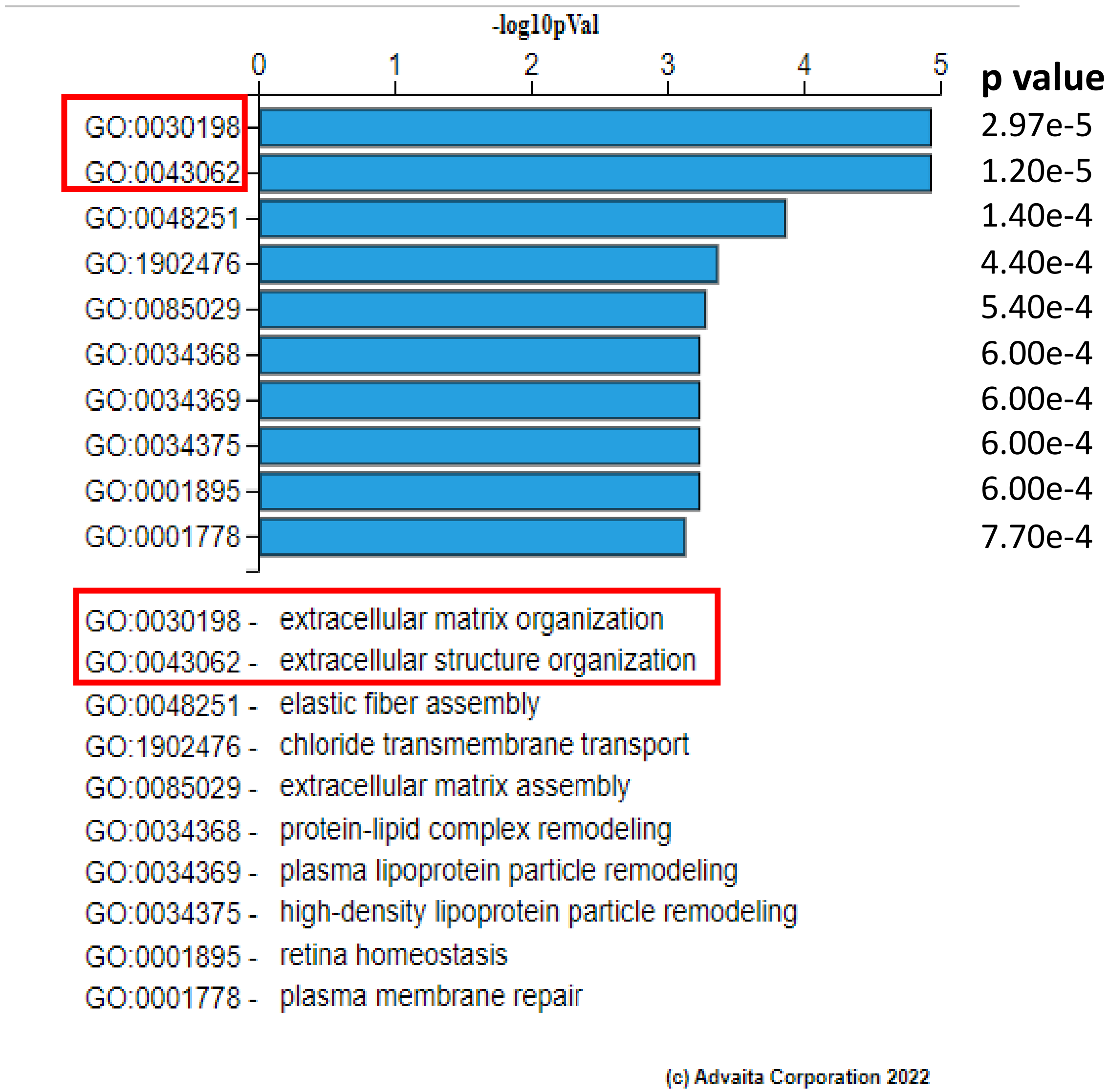

Sup. Fig. 5. Pathway analysis of RNA-seq data generated from CTRL and WAVE2-KO MDA-MB-231 cells showing the to 10 pathways that are significantly differentially dysregulated between CTRL and W2-KO cells**.** The ECM pathways rank as the top 2 pathways
