# Supplementary material for: The WAVE2/miR-29/Integrin-β1 Oncogenic Signaling Axis Promotes Tumor Growth and Metastasis in Triple-negative Breast Cancer
Source: Cancer Res Commun. 2023 Jan 31;3(1):160–74. doi: 10.1158/2767-9764.CRC-22-0249 (PMC10035451; doi:10.1158/2767-9764.CRC-22-0249)
Supplement: Supplementary Figure S6 — Western Blot analyses of WAVE2 and ITGB1 expression in different BC cell lines, and effect of loss of WAVE2 expression on levels of ITGB1, pSRC and pFAK. [file crc-22-0249-s07.pdf]

Supplementary Figure S6

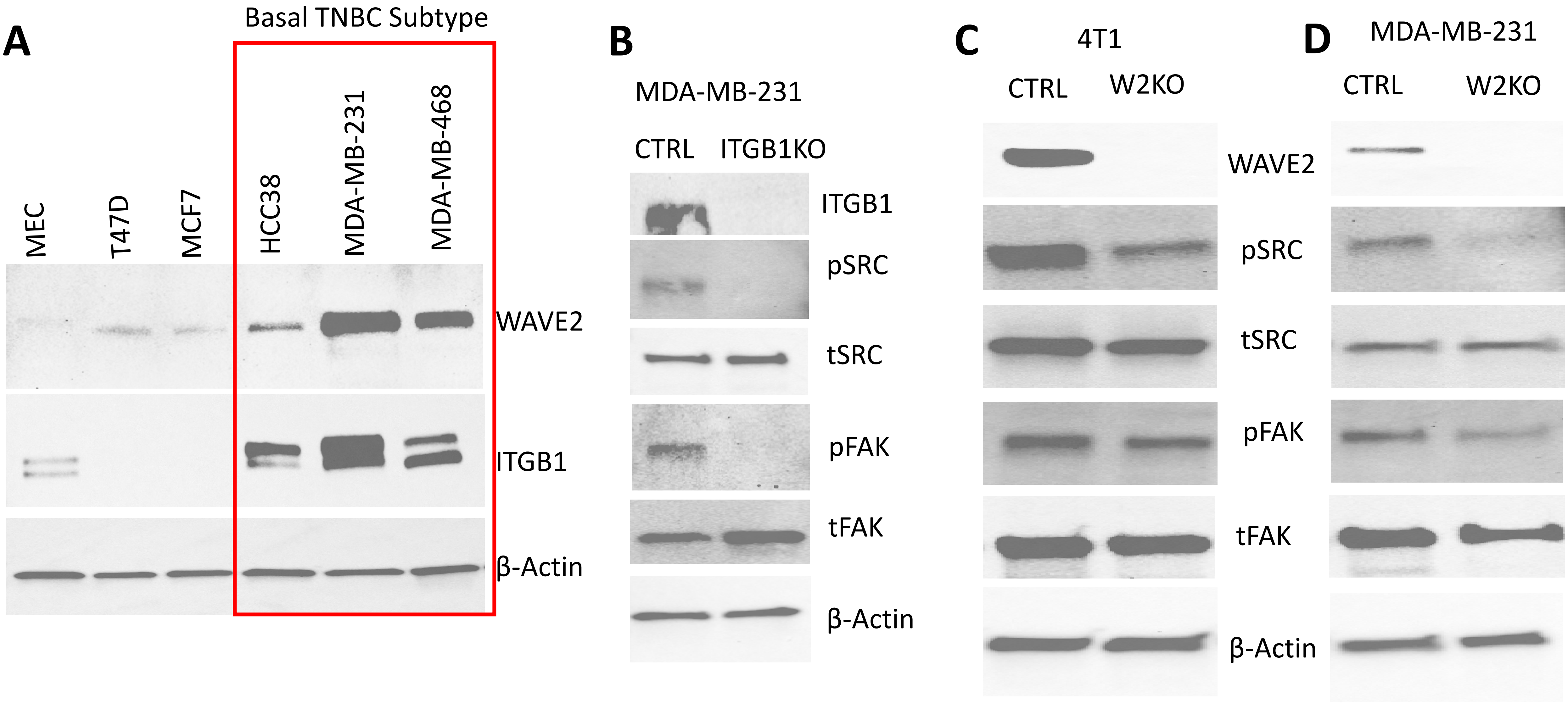

Sup. Fig. 6A. ITGB1 and WAVE2 expression levels are higher in basal TNBC cell lines.

Sup. Fig. 6B. ITGB1KO in MDA231 cells results in loss of Src and FAK phosphorylation.

Sup. Fig. 6C & D. Loss of ITGB1 expression in 4T1 (C) and 231 cells (D) as a result of W2KO leads to loss of Src and FAK phosphorylation.
