# Supplementary material for: The WAVE2/miR-29/Integrin-β1 Oncogenic Signaling Axis Promotes Tumor Growth and Metastasis in Triple-negative Breast Cancer
Source: Cancer Res Commun. 2023 Jan 31;3(1):160–74. doi: 10.1158/2767-9764.CRC-22-0249 (PMC10035451; doi:10.1158/2767-9764.CRC-22-0249)
Supplement: Supplementary Figure S7 — Target Scan prediction of micoRNA target sites in the 3'UTR of ITGB1. [file crc-22-0249-s08.pdf]

Supplementary Figure S7A

Target Scan: miR-29a/b site on ITGB-1

Human ITGB1 ENST00000396033.2 3' UTR length: 1936

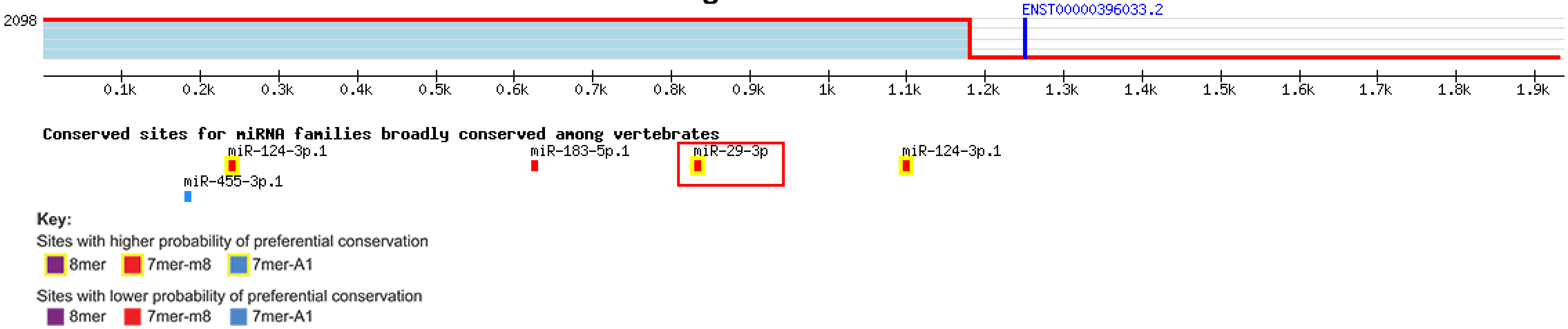

No	Position on ITGB-1	Predicted consequential pairing of target region (top) and miRNA (bottom)	Site type	Context++ score	Context++ score percentile	Weighted context++ score	Conserved branch length	P_{CT}	Predicted relative K_D
1	Position 830-836 of ITGB1 3' UTR<br>hsa-miR-29a-3p	5' ...AUUUUGUUUAAUGUC**UGGUGCU**U..<br>||| ||||||| 3' AUUGGCUAAAGUCU--**ACCACGA**U	7mer-m8	-0.36	95	-0.36	4.263	0.83	-3.242
2	Position 830-836 of ITGB1 3' UTR<br>hsa-miR-29b-3p	5' ...AUUUUGUUUAAUGUC**UGGUGCU**U.<br>||| ||||||| 3' UUGUGACUAAAGUUU--**ACCACGA**U	7mer-m8	-0.36	95	-0.36	4.263	0.83	-3.389

Sup. Fig. 7A. Target Scan prediction of microRNA seed sequences in the 3’UTR of ITGB1. Nucleotide sequence and location of the seed sequence of miR-29a and miR29b in the 3’-UTR of ITGB1 mRNA. The sequence alignment with the miR-29a and b sequences are also shown.

Supplementary Figure S7B

ITGB1 –miR29 conservation across spp

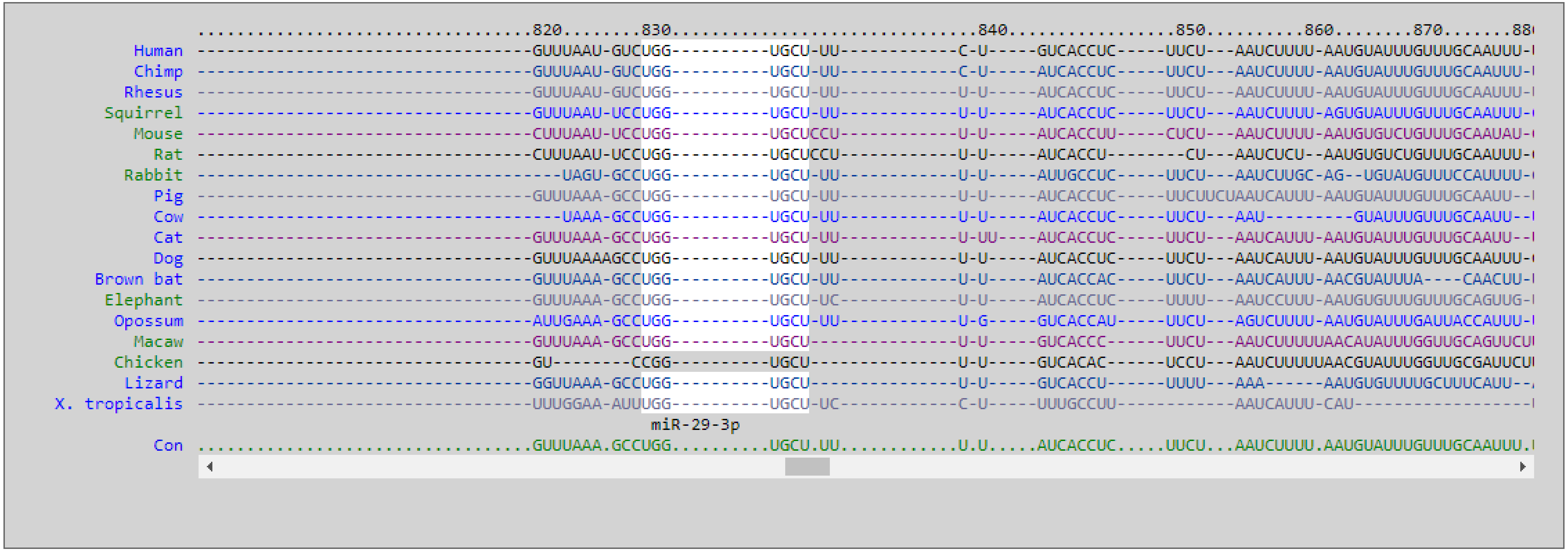

Sup. Fig. 7B. Sequence alignment of the 3’UTR of ITGB1 from different species showing the conservation of the miR-29 seed sequence across the different species
