# Supplementary material for: The WAVE2/miR-29/Integrin-β1 Oncogenic Signaling Axis Promotes Tumor Growth and Metastasis in Triple-negative Breast Cancer
Source: Cancer Res Commun. 2023 Jan 31;3(1):160–74. doi: 10.1158/2767-9764.CRC-22-0249 (PMC10035451; doi:10.1158/2767-9764.CRC-22-0249)
Supplement: Supplementary Figure S8 — Target Scan prediction of micoRNA target sites in the 3'UTR of WAVE2. [file crc-22-0249-s09.pdf]

Target Scan: miR-29a/b site on WAVE2

Supplementary Figure S8A

Human WAVE2 ENST00000536657.1 3' UTR length: 4090

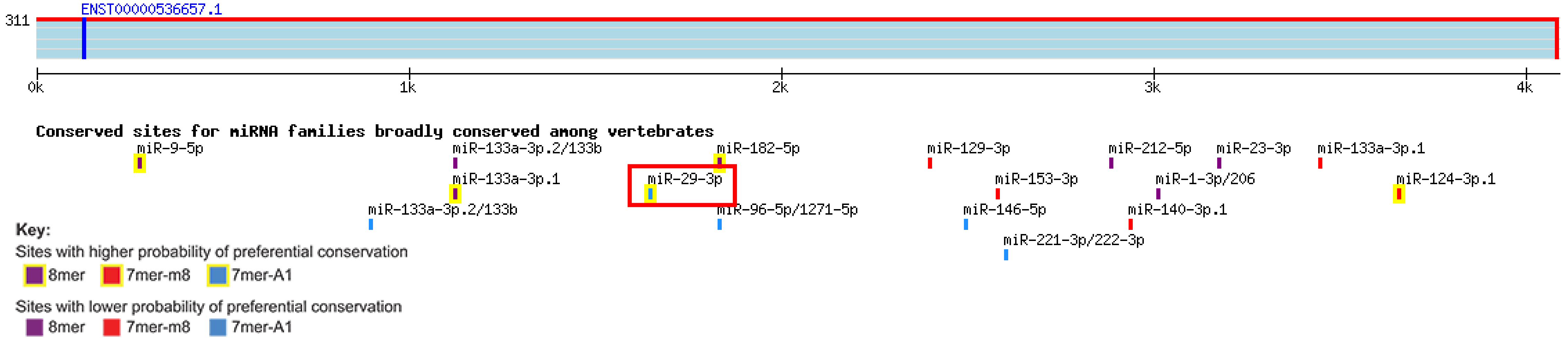

No	Position on WASF2	Predicted consequential pairing of target region (top) and miRNA (bottom)	Site type	Context++ score	Context++ score percentile	Weighted context++ score	Conserved branch length	P_{CT}	Predicted relative K_D
1	Position 1644-1650 of WAVE2 3' UTR<br>hsa-miR-29a-3p	5'...CAGAGCUAAUACACA**GGUGCUA**U...<br>|||||| 3' AUUGGCUAAAGUCUA**CCACGA**U	7mer-A1	-0.25	88	-0.24	3.858	0.79	-3.370
2	Position 1644-1650 of WAVE2 3' UTR<br>hsa-miR-29b-3p	5' ...CAGAGCUAAUACACA**GGUGCUA**U...<br>|||||| 3' UUGUGACUAAAGUUUA**CCACGA**U	7mer-A1	-0.25	88	-0.24	3.858	0.79	-3.564

Sup. Fig. 8A. Target Scan prediction of microRNA seed sequences in the 3'UTR of WAVE2. Nucleotide sequence and location of the seed sequence of miR-29a and miR29b in the 3'-UTR of WAVE2 mRNA. The sequence alignment with the miR-29a and b sequences are also shown.

WAVE2-miR29 conservation across species

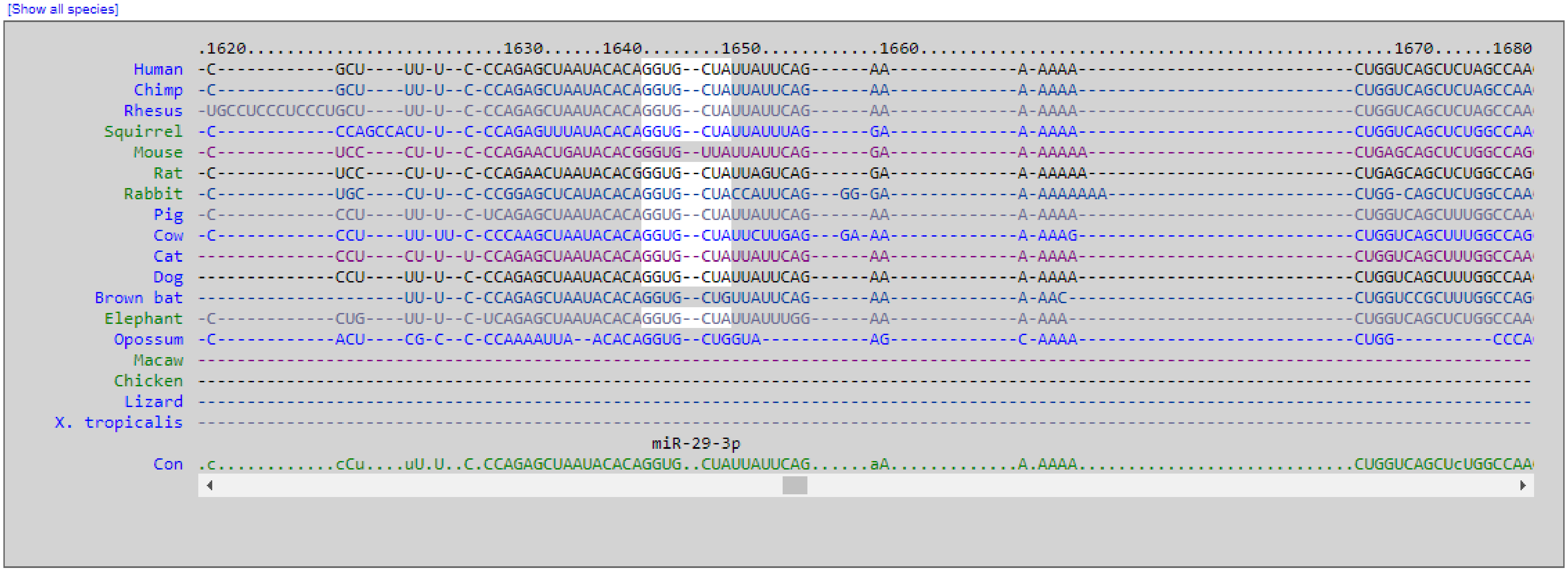

Sup. Fig. 8B. Sequence alignment of the 3'UTR of WAVE2 from different species showing the conservation of the miR-29 seed sequence across the different species
