# Supplementary material for: The WAVE2/miR-29/Integrin-β1 Oncogenic Signaling Axis Promotes Tumor Growth and Metastasis in Triple-negative Breast Cancer
Source: Cancer Res Commun. 2023 Jan 31;3(1):160–74. doi: 10.1158/2767-9764.CRC-22-0249 (PMC10035451; doi:10.1158/2767-9764.CRC-22-0249)
Supplement: Supplementary Figure S9 — Western Blot analyses confirming exogenous expression of HA-tagged WAVE2 in HEK-293 cells. [file crc-22-0249-s10.pdf]

Supplementary Figure S9

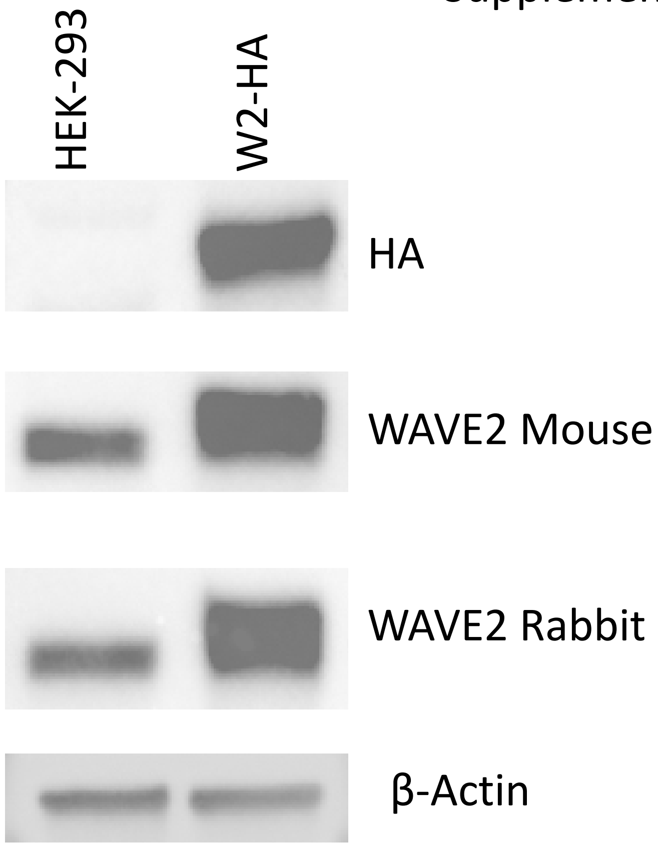

Sup. Fig. 9. Overexpression of HA-Tagged WAVE2 in HEK293 cells. Exogenous HA-W2 protein was detected using anti-HA antibody, while both endogenous WAVE2 and exogenous HA-W2 proteins were detected using both mouse-anti W2 and rabbit-antiW2 antibodies. β-Actin was used as loading control.
