# Supplementary material for: The WAVE2/miR-29/Integrin-β1 Oncogenic Signaling Axis Promotes Tumor Growth and Metastasis in Triple-negative Breast Cancer
Source: Cancer Res Commun. 2023 Jan 31;3(1):160–74. doi: 10.1158/2767-9764.CRC-22-0249 (PMC10035451; doi:10.1158/2767-9764.CRC-22-0249)
Supplement: Supplementary Figure S10 — Volcano plot from the RNA-seq analysis of the differentially expressed genes between CTRL and W2-KO MDA-MB-231 cells. [file crc-22-0249-s11.pdf]

Supplementary Figure S10

A

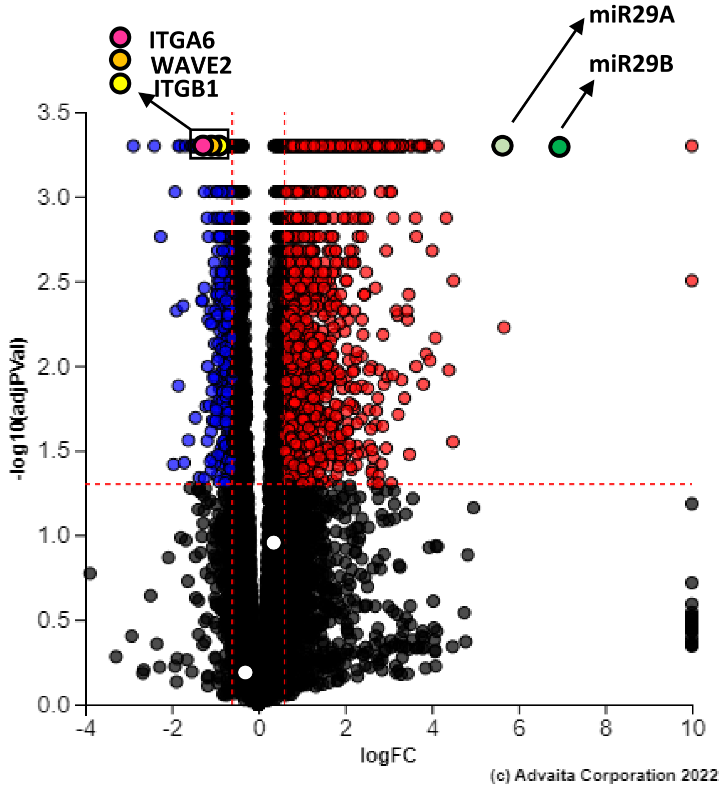

B

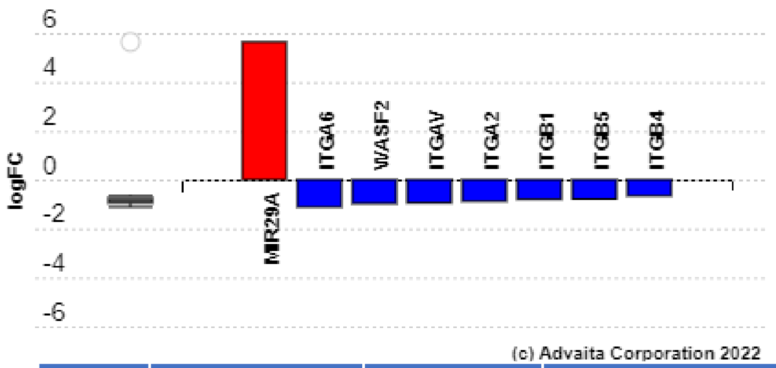

C

No.	Gene	logfc	p-value
1	WASF2	-0.972	5.031e-4
2	miR29A	+5.637	5.031e-4
3	ITGB1	-1.119	5.031e-4
4	ITGA6	-0.804	5.031e-4
5	ITGA2	-0.886	5.00E-05
6	ITGAV	-0.950	5.00E-05
7	ITGB5	-2.715	5.00E-05
8	ITGB4	-0.663	0.01035

Sup. Fig. 10. (A) Volcano plot from the RNA-seq analysis of the differentially expressed genes between CTRL and W2-KO MDA-MB-231 cells. The X-axis represents the Log2 fold change in expression levels and the Y-axis shows the p values. Red dots: upregulated genes; Blue dots: downregulated genes: black dots: no significant change. The dots of the genes of interest are labelled. Bars (B) and values (C) representation of the log2 change in expression levels of WAVE2, ITGB1, ITGA6, miR29a and miR29b derived from the RNA-seq data.
