# Supplementary material for: The WAVE2/miR-29/Integrin-β1 Oncogenic Signaling Axis Promotes Tumor Growth and Metastasis in Triple-negative Breast Cancer
Source: Cancer Res Commun. 2023 Jan 31;3(1):160–74. doi: 10.1158/2767-9764.CRC-22-0249 (PMC10035451; doi:10.1158/2767-9764.CRC-22-0249)
Supplement: Supplementary Figure S11 — Analysis of the potential role of DGCR8 in the WAVE2-mediated regulation of miR-29 in BC. [file crc-22-0249-s12.pdf]

A

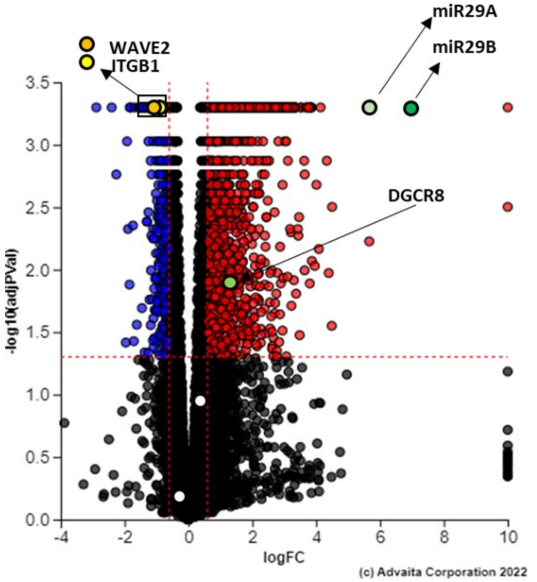

B

Gene	logfc	p-value
WASF2	-0.972	5.031e-4
miR29A	+5.637	5.031e-4
ITGB1	-1.119	5.031e-4
DGCR8	1.299	0.00195

Sup. Fig. 11A & B. (A) Volcano plot from the RNA-seq analysis of the differentially expressed genes between CTRL and W2-KO MDA-MB-231 cells., showing increased expression of DCGR8 in the W2KO cells. (B) value representation of the log2 change in expression levels of WAVE2, ITGB1, miR29a, miR29b and DGCR8 derived from the RNA-seq data

C

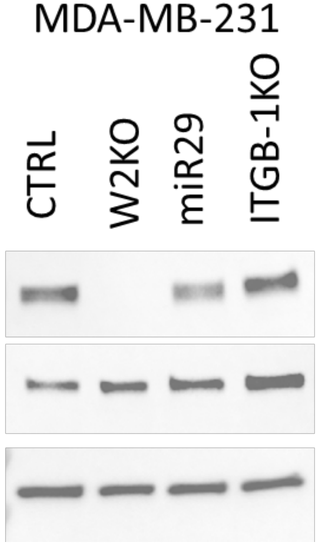

Sup. Fig. 11C. Loss of WAVE2 (W2KO) or overexpression of miR29 in MDA-MB-231 cells results in increased expression of DGCR8.

D

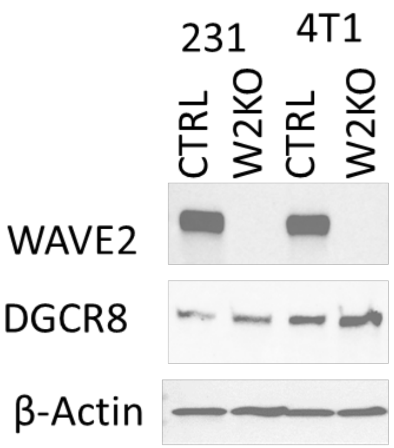

Sup. Fig. 11D. Loss of WAVE2 (W2KO) in MDA-MB-231 or 4T1 cells results in increased expression of DGCR8.

E

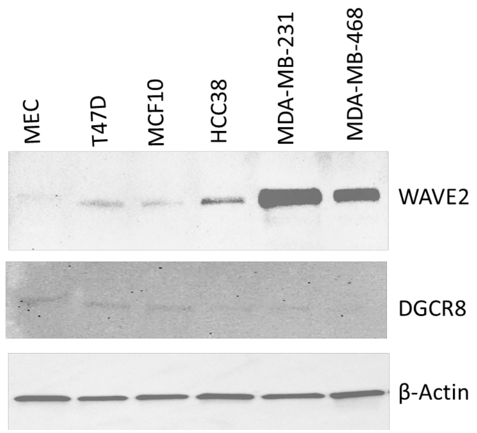

Sup. Fig. 11E. DGCR8 and WAVE2 expression in basal TNBC cell lines. The WAVE2 and Actin panels are duplicated from Sup. Fig. 6A and a re shown here again for clarity.

F

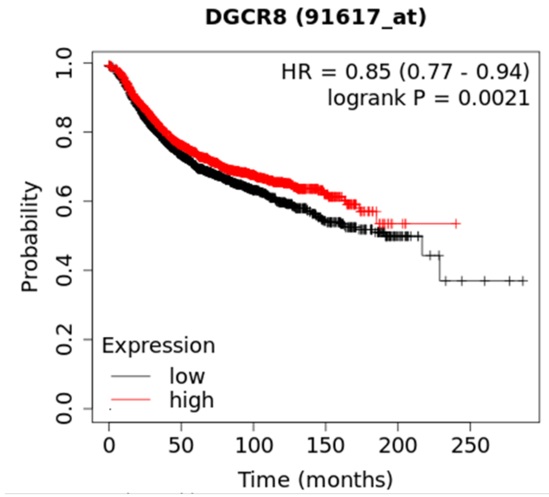

Low expression cohort (months)	High expression cohort (months)
45.44	55.2

Sup. Fig. 11E. KM plots correlating survival of 4929 BC patients with DGCR8 mRNA expression levels. High DGCR8 expression levels correlate with poor survival probability in BC patients (p = 0.0021 and p=0.00049). Number of patients at risk and median survival in the low and high WAVE2 cohorts are also shown

Supplementary Figure S11
