# Supplementary material for: The WAVE2/miR-29/Integrin-β1 Oncogenic Signaling Axis Promotes Tumor Growth and Metastasis in Triple-negative Breast Cancer
Source: Cancer Res Commun. 2023 Jan 31;3(1):160–74. doi: 10.1158/2767-9764.CRC-22-0249 (PMC10035451; doi:10.1158/2767-9764.CRC-22-0249)
Supplement: Supplementary Figure S12 — Immunofluorescence and Western Blot analyses of WAVE2 distribution between the cytoplasmic and the nuclear compartments of MDA-MB-231 cells. [file crc-22-0249-s13.pdf]

Supplementary Figure S12

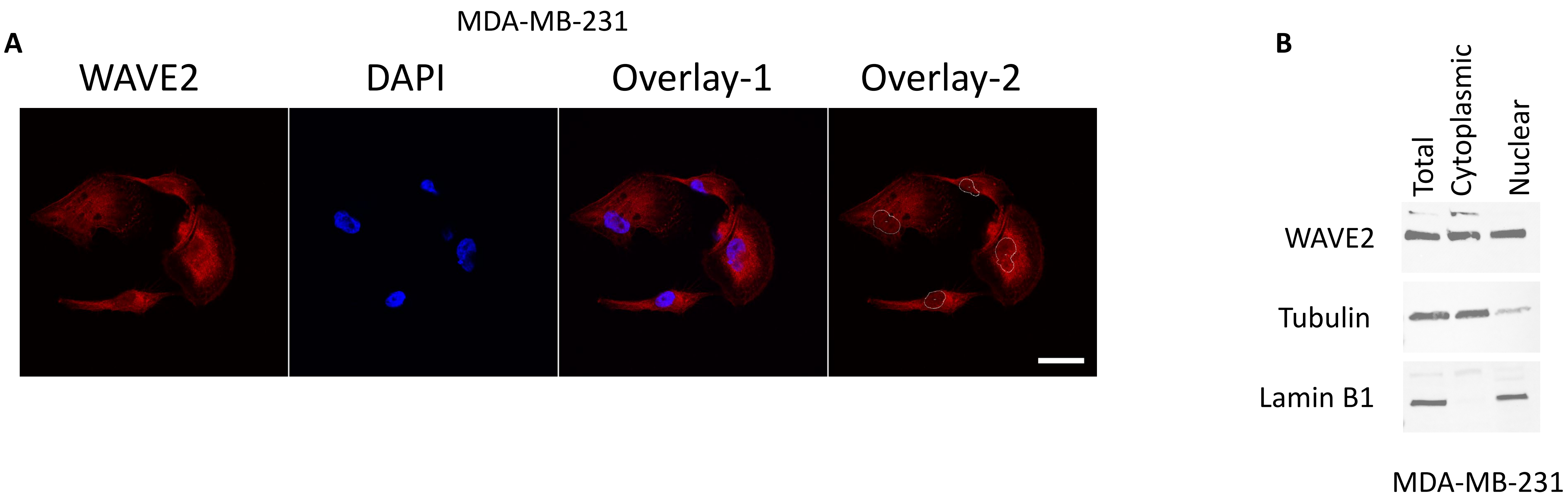

Sup Fig. 12. (A) Confocal microscopy images of immunofluorescence staining of MDA-MB-231 cells that were stained for WAVE2 (red). Nuclei were counterstained with DAPI. (B) Representative Western blot of protein lysates; total, cytoplasmic and nuclear fraction, probed with anti-WAVE2-antibody. Tubulin and Lamin B1, were used as positive controls for the cytoplasmic and the nuclear fraction, respectively.
