# Supplementary material for: The WAVE2/miR-29/Integrin-β1 Oncogenic Signaling Axis Promotes Tumor Growth and Metastasis in Triple-negative Breast Cancer
Source: Cancer Res Commun. 2023 Jan 31;3(1):160–74. doi: 10.1158/2767-9764.CRC-22-0249 (PMC10035451; doi:10.1158/2767-9764.CRC-22-0249)
Supplement: Supplementary Figure S13 — Immunofluorescence analysis of the effect of loss of WAVE2 on lamellipodia formation in MDA-MB-231 cells. [file crc-22-0249-s14.pdf]

Supplementary Figure S13

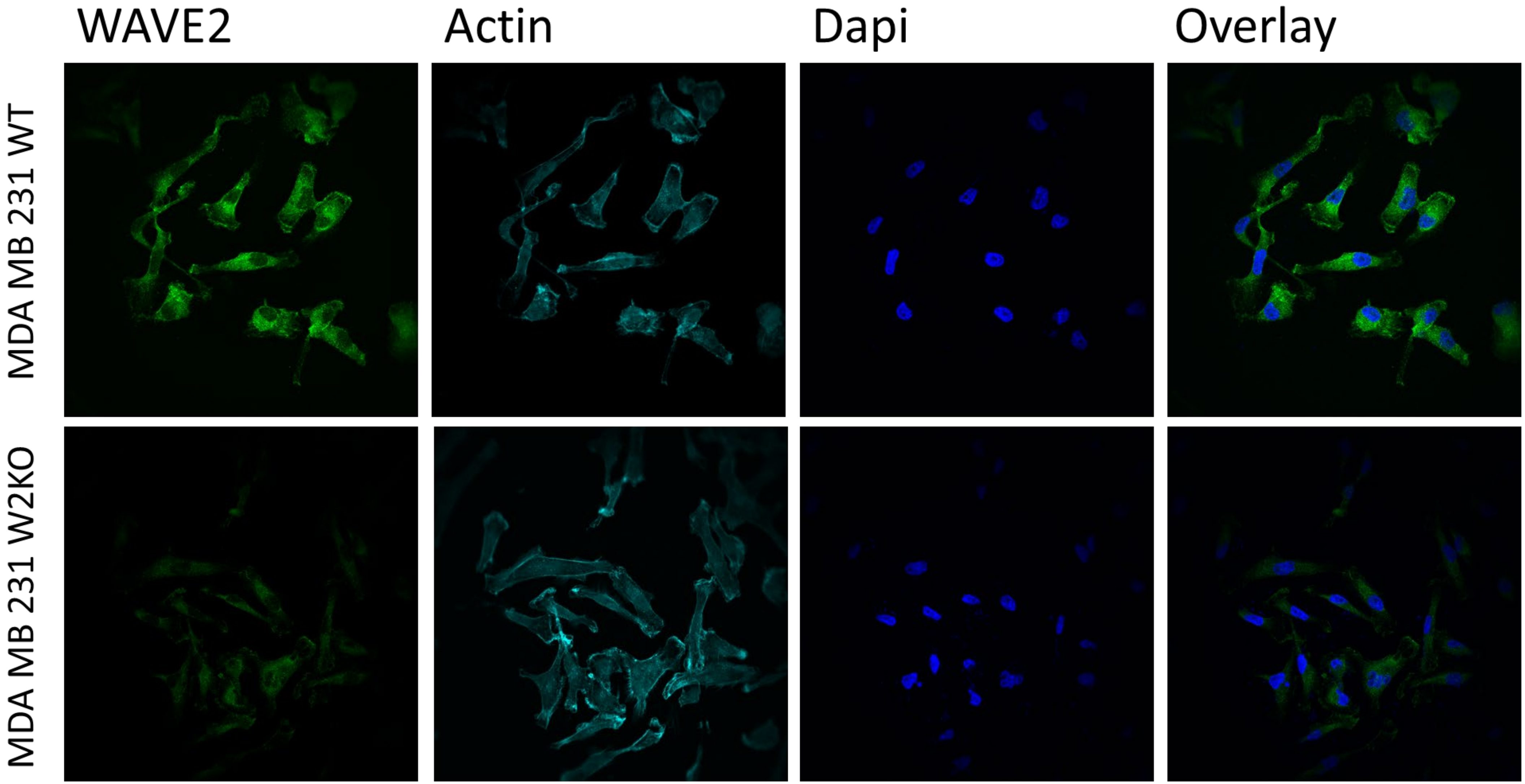


**Sup. Fig. 13: Loss of WAVE2 affects lamellipodia formation at the leading edge**: Confocal microscopy images of immunofluorescence staining of MDA-MB-231 or their W2KO derivatives cells that were stained for WAVE2 (Green), and actin (Magenta). Nuclei were counterstained with DAPI.
